# Exploration of the synergistic effect of chrysene-based core and benzothiophene acceptors on photovoltaic properties of organic solar cells

**DOI:** 10.1038/s41598-024-65459-6

**Published:** 2024-07-02

**Authors:** Iqra Shafiq, Shehla Kousar, Faiz Rasool, Tansir Ahamad, Khurram Shahzad Munawar, Saifullah Bullo, Suvash Chandra Ojha

**Affiliations:** 1 Institute of Chemistry, Khwaja Fareed University of Engineering & Information Technology, Rahim Yar Khan, 64200 Pakistan; 2 Centre for Theoretical and Computational Research, Khwaja Fareed University of Engineering & Information Technology, Rahim Yar Khan, 64200 Pakistan; 3 Institute of Chemical Sciences, Bahauddin Zakariya University, Multan, 60800 Pakistan; 4 Department of Chemistry, King Saud University, 11451 Riyadh, Saudi Arabia; 5 Institute of Chemistry, University of Sargodha, Sargodha, 40100 Pakistan; 6 Department of Chemistry, University of Mianwali, Mianwali, 42200 Pakistan; 7 Department of Human and Rehabilitation Sciences, Begum Nusrat Bhutto Women University, Sukkur Sindh, Pakistan; 8 Department of Infectious Diseases, The Affiliated Hospital of Southwest Medical University, Luzhou, 646000 China

**Keywords:** NF-OSCs, Photovoltaic response, Chrysene derivatives, Open circuit voltage, Binding energy, Chemistry, Materials science, Optics and photonics

## Abstract

To improve the efficacy of organic solar cells (OSCs), novel small acceptor molecules (**CTD1–CTD7**) were designed by modification at the terminal acceptors of reference compound **CTR**. The optoelectronic properties of the investigated compounds (**CTD1–CTD7**) were accomplished by employing density functional theory (DFT) in combination with time-dependent density functional theory (TD-DFT). The M06 functional along with a 6-311G(d,p) basis set was utilized for calculating various parameters such as: frontier molecular orbitals (FMO), absorption maxima (*λ*_max_), binding energy (*E*_b_), transition density matrix (TDM), density of states (DOS), and open circuit voltage (*V*_*oc*_) of entitled chromophores. A red shift in the absorption spectra of all designed chromophores (**CTD1–CTD7**) was observed as compared to **CTR**, accompanied by low excitation energy. Particularly, **CTD4** was characterized by the highest *λ*_max_ value of 685.791 nm and the lowest transition energy value of 1.801 eV which might be ascribed to the robust electron-withdrawing end-capped acceptor group. The observed reduced binding energy (Eb) was linked to an elevated rate of exciton dissociation and substantial charge transfer from central core in HOMO towards terminal acceptors in LUMO. These results were further supported by the outcomes from TDM and DOS analyses. Among all entitled chromophores, **CTD4** exhibited bathochromic shift (685.791 nm), minimum HOMO/LUMO band gap of 2.347 eV with greater CT. Thus, it can be concluded that by employing molecular engineering with efficient acceptor moieties, the efficiency of photovoltaic materials could be improved.

## Introduction

Over the last decade, significant advancements have been achieved in organic solar cells (OSCs) on account of enhanced insights into device optimization, molecular design, and operational mechanisms^1^. Organic solar cells consist of organic semiconductors acting as electron donors and acceptors^2^. Despite the substantial development of donor materials over three decades, a limited number of acceptors have historically demonstrated the ability to achieve high power conversion efficiencies^3^. Non-fullerene acceptor (NFA) molecules have emerged as an auspicious and competent category of compounds, characterized by high efficiencies, low band gaps, tunable energy levels, and strong UV–Vis absorption^4^. The development of NFAs mark a transformative leap in cell efficiencies, with the notable achievement of reaching the significant milestone of 20%^5,6^. Recently, modifying the chemical structure has proven to be a successful strategy for adjusting the photovoltaic characteristics of non-fullerene compounds. This modification involves altering the side chains, end-capped acceptor units, and the core molecule^7,8^. Functionalizing the end groups (EG) of central donor core with non-fullerene acceptors (NFA) demonstrated to be an effective approach for boosting the performance of organic solar cells (OSCs)^7–9^. This, in turn, plays a substantial role in shaping the power conversion efficiency (PCE) and various other performance metrics of NFA-based OSCs (NF-OSCs)^9^. The A–*π*–A architecture, consisting of central electron-donating *π*-spacer unit combined with two electron-deficient end-capped acceptor groups on both sides is considered as an effective approach in NF–OSCs to improve intramolecular charge transfer (ICT)^10^. A novel electron-donating core, formed by condensing chrysene with two thiophenes via two dihydrobenzene rings, was synthesized by Lu and his coworkers. Utilizing this core along with two electron-accepting end groups of 1,1-dicyanomethylene-3-indanone, a new Z-shaped fused-ring electron acceptor was developed and synthesized. This chrysene-based compound exhibited strong absorption within the 500–850 nm range, a bandgap measuring 1.50 eV, and a charge mobility of 2.5 × 10^−4^ cm^2^ V^−1^ s^−1^ ^11^. In another report, chrysene based compound with PCE value upto 24.7% on blending with poly (3-hexylthiophene) (P_3_HT) and phenyl-C61-butyric acid methyl ester (PCBM) polymers was stated^12^. Moreover, in a previous study, a chrysene-core based derivative with *V*_*oc*_ value of 1.20 V has been reported^13^.

Encouraged by findings, we developed A–*π*–A type seven novel chrysene-based, non-fullerene, fused-ring electron acceptor materials (**CTD1–CTD7**). The designing of reference compound **CTR** has been carried out by the insertion of highly efficient benzothiophene end-capped acceptor at the peripherals of a recently synthesized parent molecule **CTIC-4F**^24^. The DFT study of compounds containing chrysene core fused with benzodithiophene terminal acceptors has not been reported yet. Introducing the electron-proficient benzothiophene unit into the end-group raises the energy levels of the lowest unoccupied molecular orbitals (LUMO) in non-fullerene acceptors (NFAs), resulting in increased *V*_*oc*_ outputs. Moreover, it encourages antiparallel π–π stacking of the end-groups, thereby facilitating efficient electron transport^14^. The reference compound was further fabricated to design **CTD1–CTD7** derivatives by introducing various electrophilic groups in to the benzothiophene acceptors. The newly designed molecules **CTD1–CTD7** possess unique characteristics attributable to the presence of diverse groups and atoms in their terminal acceptor regions. Hence, employing various electrophilic groups leads to the formation of more efficient OSC molecules from chrysene core.

To assess the photovoltaic and optoelectronic properties of these materials, DFT and TD-DFT calculations have been employed. Analyses including frontier molecular orbital (FMO), density of states (DOS), transition density matrix (TDM), binding energy (*E*_b_), and open circuit voltage (*V*_oc_) have been conducted to elucidate the electronic, photophysical, photovoltaic, and charge transfer characteristics of the reference molecule **CTR** and its designed compounds (**CTD1–CTD7**). Noticeably, all the designed derivatives showed good *V*_*oc*_ values, revealing their potential for paramount current generation capacity. Thus, it is highly anticipated that these newly designed compounds (**CTD1–CTD7**) will play a remarkable role in advancing the development of highly efficient organic solar cell (OSC) materials.

## Computational procedure

**Figure 1:**
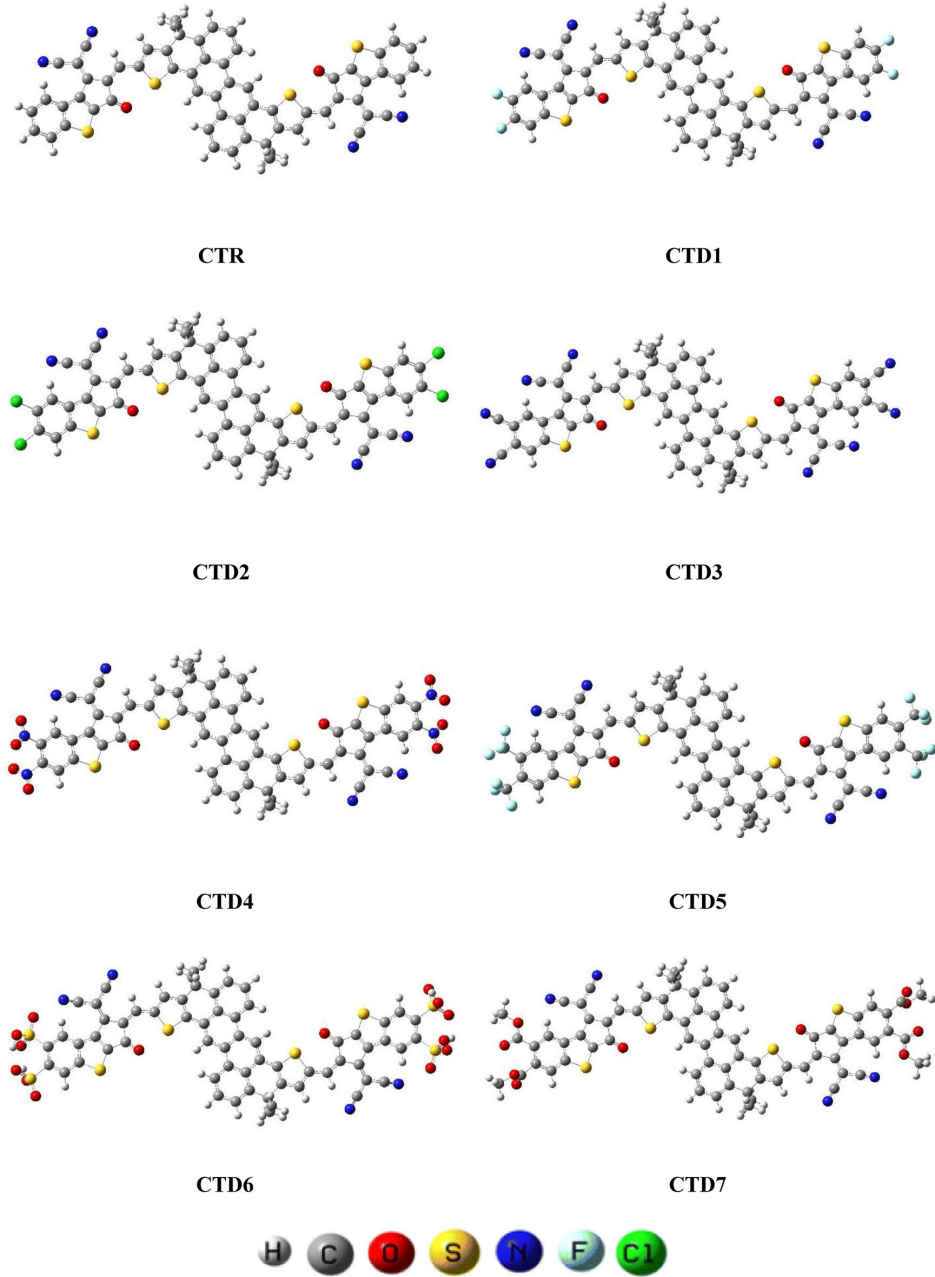
Optimized structures of **CTR** tailored chromophores **CTD1–CTD7.**

The computational investigation involving density functional theory (DFT) and time-dependent density functional theory (TD-DFT) were carried out using the Gaussian 09^15^ software package, with visualization of results facilitated by GaussView 6.0^16^. The M06 ^17^ coupled with the 6-311G(d,p) basis set was employed to perform all analyses. At first geometrical optimization was accomplished to get true minima structures. By utilizing these optimized structures, various photovoltaic and optoelectronic parameters such as frontier molecular orbitals (FMOs), transition density matrix (TDM), global reactivity descriptors (GRDs), density of state graphs (DOS), binding energy (*E*_*b*_), and open circuit voltage (*V*_*oc*_) were computed at the entitled functional. Multiple software like Avogadro^18^, Multiwfn^19^, PyMOlyze^20^, GaussSum^21^, Origin^22^ and Chemcraft^23^ were used to interpret data in tabular and and different graphs (Fig. 1).

## Results and discussion

**Figure 2:**
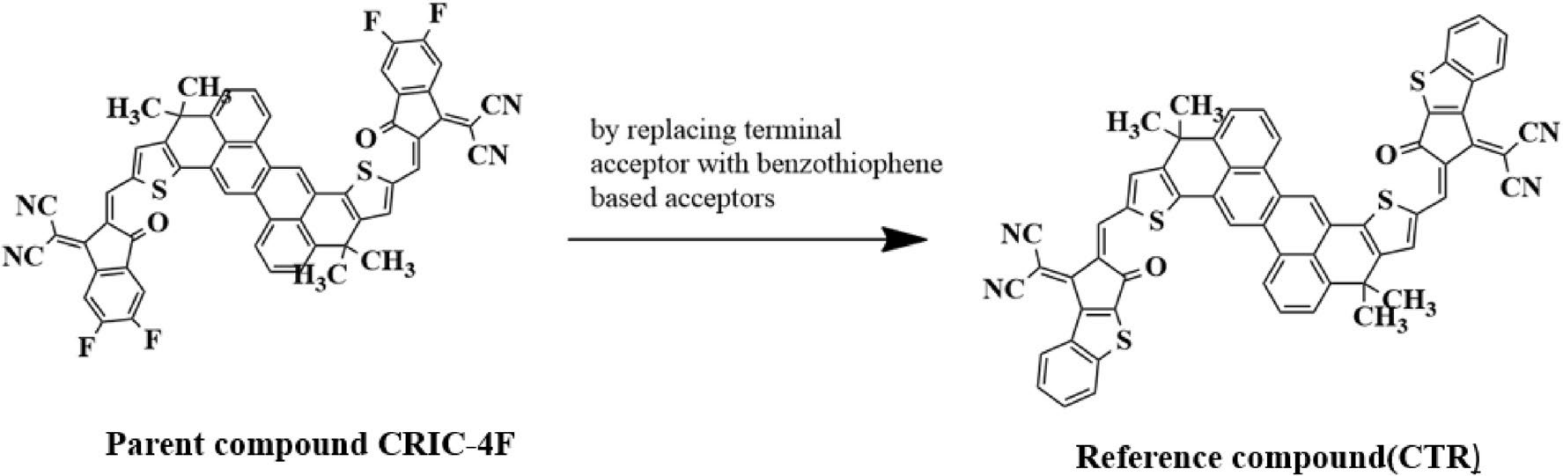
Designing of reference molecule (**CTR**) from parent chromophore.

Inspiring from the research of Zhao et al.^24^, we have designed a series of small organic molecular non-fullerene acceptors (NFAs) from parent compound **CTIC-4F** categorized as **CTR** and **CTD1–CTD7** with A–*π*–A architecture, incorporating a 4,4,12,12-tetramethyl-4,12-dihydrodibenzo[4,5:10,11]tetraceno[1,2-b:7,8-b']dithiophene *π*-spacer moiety. The terminal acceptor group of the parent molecule was substituted with benzothiophene-based acceptor to design the reference chromophore (**CTR**), as depicted in Fig. 2.

**Figure 3:**
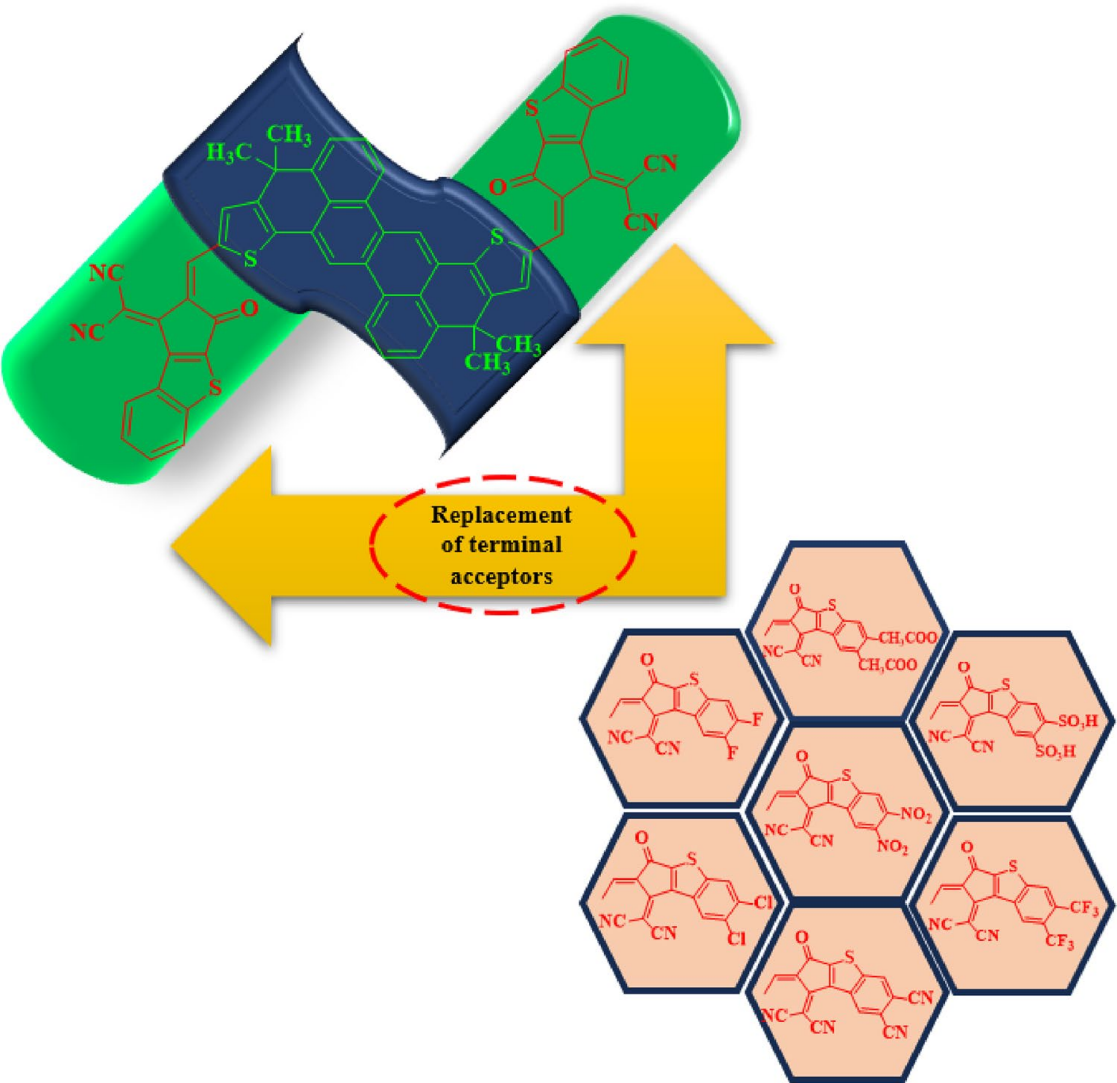
Schematic representation of designing of derivatives (**CTD1–CTD7**) from reference (**CTR**).

The peripheral electron accepting units of **CTR** were systematically substituted with diverse electron-deficient groups with the objective of formulating efficient non-fullerene OSCs. Through the substitution of terminal groups like –F, –Cl, –CN, –NO2, –CF3, –SO3H, and –CH3COO, seven distinct derivatives **CTD1, CTD2, CTD3, CTD4, CTD5, CTD6** and **CTD7** were designed, respectively (Fig. 3). The IUPAC names and 2D-structures of these derivatives are arranged in Table S21, while the optimized structures of designed derivatives are presented in Fig. 1. The Cartesian coordinates of investigated compounds are portrayed in Tables S22–S29.

The primary objective of this work is to design NFAs with enhanced photovoltaic properties. In this study, our focus is on designing new NFAs that exhibit significantly improved photovoltaic characteristics, including a narrow optical bandgap, low excitation energy and increased absorption. The theoretically simulated parameters aim to facilitate the development of molecules with superior properties for solar cell applications.

## Electronic studies

Investigating the FMOs provides valuable insights into a molecule's reactivity, helping establish the active site through orbital distribution^25^. The determination of effective charge transfer across donor and acceptor components in photovoltaic devices heavily relies on assessing the energy levels of the highest occupied molecular orbital (HOMO) and the lowest unoccupied molecular orbital (LUMO)^26–29^. Analyzing the HOMO–LUMO relationship is essential for exploring quantum chemical properties. This study aids in predicting reactive sites within π-electron systems and offers explanations for various reactions within conjugated frameworks^30^. The energy band gap in frontier molecular orbitals (FMOs) plays a crucial role in molecular modeling, influencing dynamic structural stability, charge generation, electrical conductivity, chemical reactivity, short-circuit current (*J*_sc_), power conversion efficiency (PCE) and open-circuit voltage (*V*_*oc*_)^31,32^. A smaller band gap (*E*_g_) in FMOs facilitates the flow of charge from donor’s HOMO and acceptor’s LUMO, facilitating current generation^33^. FMO analysis on reference compound **CTR** and designed derivatives **CTD1–CTD7** has been conducted, utilizing the M06 level of theory in conjunction with the 6-311G(d,p) basis set. Figure 2 illustrates the alignment of FMOs of **CTR** and **CTD1–CTD7**. Table 1 represents energies of HOMO–LUMO and their respective energy gaps.

**Table 1:** Energies of frontier molecular orbitals of the **CTR** and **CTD1–CTD7.**

Compound	HOMO	LUMO	*Eg*
CTR	− 5.772	− 3.279	2.493
CTD1	− 5.816	− 3.348	2.468
CTD2	− 5.833	− 3.377	2.456
CTD3	− 5.938	− 3.534	2.404
CTD4	− 5.950	− 3.578	2.327
CTD5	− 5.885	− 3.443	2.442
CTD6	− 5.946	− 3.546	2.400
CTD7	− 5.834	− 3.383	2.451

Band gap = *E*_LUMO_ − *E*_HOMO_, Units in *eV.*

Table 1 illustrates the HOMO and LUMO energies for reference molecule **CTR** as − 5.772/− 3.279 eV corresponding energy gap of 2.493 eV. In comparison, all derivatives (**CTD1–CTD7**) exhibited HOMO/LUMO energies of − 5.816/− 3.348, − 5.833/− 3.377, − 5.938/− 3.534, − 5.950/− 3.578, − 5.885/− 3.443, − 5.946/− 3.546, and − 5.834/− 3.383 eV, along with energy gaps of 2.468, 2.456, 2.404, 2.327, 2.442, 2.400, and 2.451 eV, respectively. The decreasing energy gap observed in these derivatives can be ascribed to the successive substitution of robust electron-withdrawing moieties, increasing acceptor strength and enhancing the charge transference as compared to **CTR**. It is worth noting that the intramolecular charge transfer (ICT) of a compound is inversely proportional to energy band gap. A minor energy gap indicates better transfer of charge and vice versa^34^. General decreasing sequence of band gap is given as: **CTR** > **CTD1** > **CTD2** > **CTD7** > **CTD5** > **CTD3** > **CTD6** > **CTD4** with *E*_*g*_ values 2.493, 2.468, 2.456, 2.451, 2.442, 2.404, 2.400, and 2.327 eV, respectively. Thus, **CTD4** chromophore represented an effective charge mobility across terminal acceptors through the chrysene donor by showing the lowest energy gap between the molecular orbitals, making it an efficient candidate for applications in photovoltaic devices.

**Figure 4:**
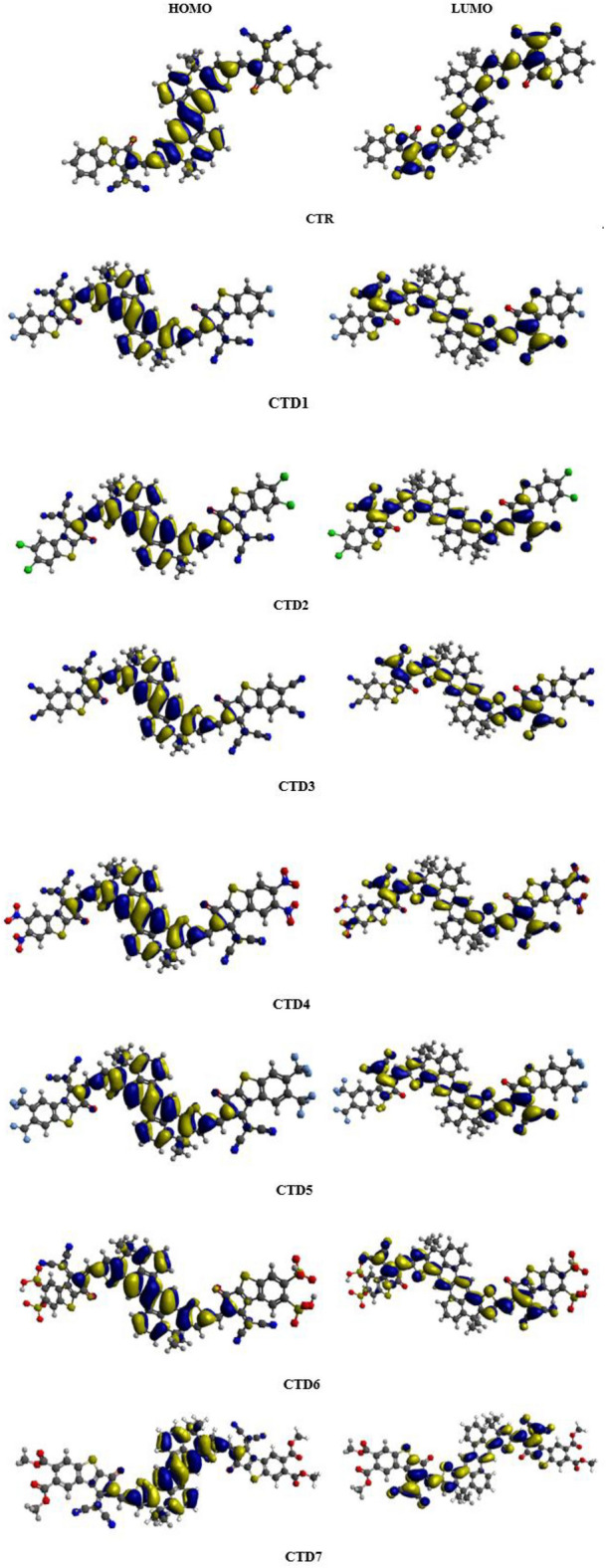
Energy Orbital diagrams of **CTR** and designed chromophores **CTD1–CTD7**.

Figure 4 portrays surface diagrams of the FMOs, showcasing the spreading of electronic density clouds across molecules. For all the designed compounds (except **CTD4**), there is a significant concentration of charge density in the central part in HOMO, with a minor presence of electronic cloud observed over the end-capped acceptor groups in LUMO. For **CTD4**, the HOMO density is predominantly intense on the *π*-bridge, but the LUMO density is highly concentrated in the nitro group among electron-deficient end-capped groups. Consequently, compounds under analysis exhibited ICT from one terminal acceptor to the other across the *π*-core. HOMO − 1/LUMO + 1 and HOMO − 2/LUMO + 2 energies are detailed in Tables S1 and S2 respectively, and their corresponding FMO diagrams are presented in Fig. S1. Similar phenomena in terms of energies and charge transfer are observed among above level orbitals (HOMO − 1/LUMO + 1 and HOMO − 2/LUMO + 2). The GRDs of entitled chromophores are tabulated Table S3.

## Density of states (DOS)

**Figure 5:**
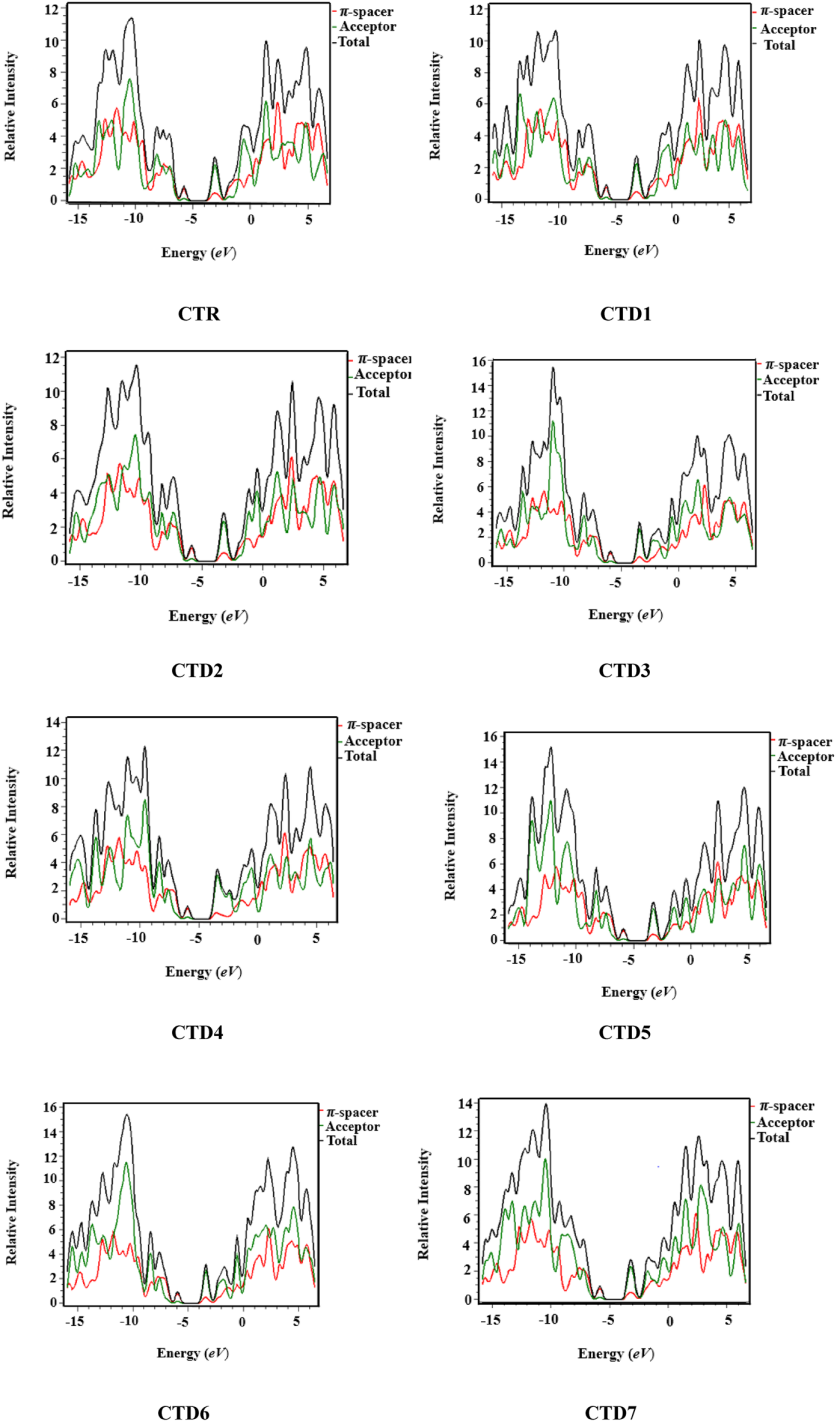
Density of states (DOS) plots for compounds **CTR** and **CTD1–CTD7.**

DOS study is employed to determine the distinct contribution of each fragment within the molecular system^35^. This study reveals the distribution of charge, indicating that HOMO possesses a significant capacity to donate electrons, while LUMO exhibits capability to accept electrons^36,37^. The distribution of electronic charges varies with different acceptor units, as evidenced by the HOMO/LUMO percentage of density of states (DOS) presented in Table S4. In this context, the acceptor units show contributions of electronic charges as follows: 16.5%, 16.4%, 16.4%, 16.4%, 16.5%, 16.2%, 16.4%, and 16.3% to HOMO, while 65.1%, 66.2%, 65.9%, 62.5%, 63.5%, 63.8%, 61.3%, and 66.1% to LUMO for **CTR** and **CTD1–CTD7**, respectively. Similarly, the *π*-bridge demonstrates charge distribution as follows: 83.5%, 83.6%, 83.5%, 83.6%, 83.5%, 83.8%, 83.6%, and 83.7% to HOMO, whereas 34.9%, 33.8%, 34.1%, 37.5%, 36.5%, 36.2%, 38.7%, and 33.9% to LUMO for **CTR** and **CTD1–CTD7**, respectively. From DOS graphs, LUMO is indicated by positive values, HOMO is specified by negative values along the x-axis. The energy gap (*E*_g_) expresses the distance between HOMOs and LUMOs (Fig. 5). **CTD4** DOS spectrum suggested that the maximum positive charge density (HOMO) is located over the *π*-spacer at approximately − 10.5 eV, while LUMO is primarily located on the acceptor, with its largest peak occurring at 3.5 eV. Hence, DOS analysis revealed massive transfer of charges across terminal acceptors through the *π*-bridge in all studied chromophores (**CTD1–CTD7**).

## Optical properties

Optical properties are essential for evaluating overall operational capabilities of organic solar cells (OSCs)^38,39^. The UV–Vis spectra of all the investigated molecules have been thoroughly examined using TD-DFT/M06/6-311G(d,p). Although, UV–Vis data were computed for six excited states (as presented in Tables S5–S20), only data pertaining to the first excited state are shown in Table 2. This selection is justified as the first excited state contains maximum values of wavelength of absorption (*λ*_max_), oscillator strength (*f*_os_), first excitation energy (*E*), and the highest percentage of intramolecular charge transfer (ICT).

**Table 2:** The outcomes of UV–visible absorption spectroscopy for **CTR** and **CTD1–CTD7** in both solvent and gaseous phases.

Medium	Compounds	${\varvec{\lambda}}$_max_ (nm)	*E*(*eV*)	*f*_*os*_	MO contributions
Solvent	**CTR**	651.109	1.904	2.005	H → L (94%), H-1 → L + 1 (2%)
	**CTD1**	657.497	1.886	1.979	H → L (94%), H-1 → L + 1 (2%), H → L + 2 (2%)
	**CTD2**	662.061	1.873	2.081	H → L (93%), H → L + 2 (2%)
	**CTD3**	678.510	1.827	2.228	H → L (95%),
	**CTD4**	685.791	1.808	2.210	H → L (95%),
	**CTD5**	666.295	1.861	2.094	H → L (94%), H → L + 2 (2%)
	**CTD6**	680.223	1.823	2.243	H → L (95%),
	**CTD7**	663.301	1.869	2.086	H → L (94%), H → L + 2 (2%)
Gaseous phase	**CTR**	617.913	2.007	1.797	H → L (96%) H-1 → L + 1 (2%)
	**CTD1**	625.204	1.983	1.747	H → L (96%)
	**CTD2**	628.627	1.972	1.882	H → L (96%)
	**CTD3**	643.840	1.926	2.031	H → L (97%)
	**CTD4**	647.606	1.915	2.027	H → L (97%)
	**CTD5**	632.863	1.959	1.901	H → L (96%)
	**CTD6**	644.643	1.923	2.092	H → L (97%)
	**CTD7**	628.819	1.972	1.915	H → L (96%)

*f*_*os*_ = oscillator strength, H = HOMO, L = LUMO, MO = molecular orbital

**Figure 6:**
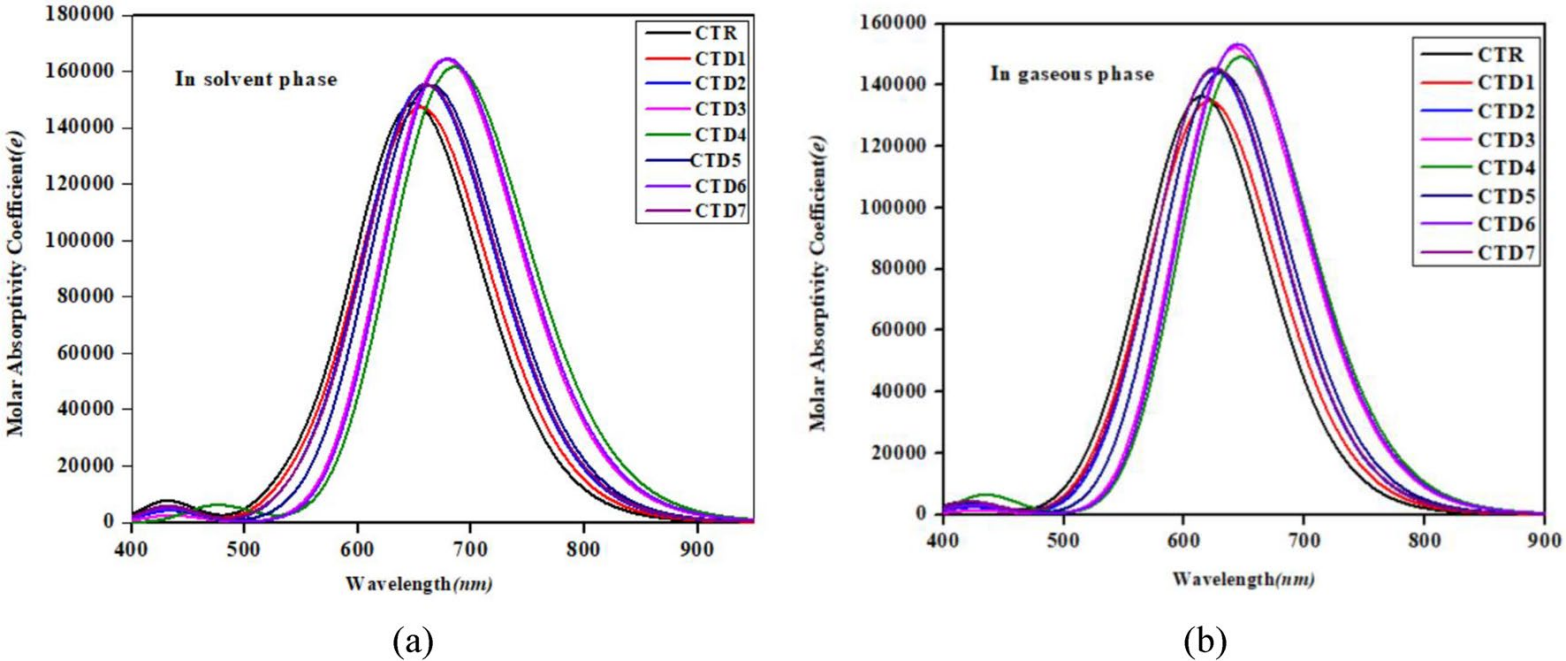
UV–Vis absoption spectra of designed compounds (**a**) in solvent phase and (**b**) in gaseous phase.

UV–Vis spectra for both the solution and gas phases are illustrated in Fig. 6. In the gaseous phase, the reference **CTR** and designed molecules **CTD1–CTD7** exhibited a red shift in absorbance. But the values of maximum absorption in the chloroform solvent are shifted more bathochromically as a consequence of solvent effect. The optical properties of a compound are also substantially influenced by its internal morphology. The interaction of π–π conjugation between the rings of end groups is crucial in determining intermolecular electronic couplings and facilitating charge transport^40,41^. Moreover, as the electron-pulling capacity of the end groups increases, the intramolecular charge transfer (ICT) between the core and end groups strengthens, resulting in a red-shift in the absorption^42^. The **CTR** exhibited the *λ*_max_ of 651.109 nm by showing oscillator strength and excitation energy and at 2.005 and 1.904 eV, respectively. Increase in absorption wavelength is observed as **CTD1** (657.497 nm), **CTD2** (662.061 nm), **CTD7** (663.301 nm) and **CTD5** (666.295 nm). This increase in wavelength can be attributed to the robust electrophilic nature of their end-capped acceptors. Moreover, a bathochromic shift is also observed in compounds **CTD3** (678.510 nm) and **CTD6** (680.223 nm). Interestingly, **CTD4** showed the maximum results of *λ*_max_ (685.791 nm) accompanying with minimum *E* (1.8808 eV) owing to the incorporation of powerful electron-pulling nitro groups in end-capped acceptor moieties. Decreasing trend of *λ*_max_ in the gaseous phase for all compounds can be found as follows: **CTD4 > CTD6 > CTD3 > CTD5 > CTD7 > CTD2 > CTD1 > CTR**. The increase in absorption spectra is investigated as the electron withdrawing efficacy of moieties increased over the terminal acceptors. Remarkably, exactly similar trend of *λ*_*max*_ in the solvent phase has been observed by proving **CTD4** as excellent organic material for obtaining maximum efficiency of solar cell materials.

Greater the first molecular orbital (MO) contributions, higher the absorbance wavelength of compounds. The results clearly indicate that excitations originating from the first excited states, specifically HOMO → LUMO transitions, predominantly contribute (between 93 and 97%) to the absorption maxima observed in the designed compounds, regardless of whether they are in solvent or gas phases. It is noteworthy that molecules in which H → L transitions highly contribute tend to have higher *λ*_*max*_ values. Therefore, **CTD3**, **CTD4**, and **CTD6** holding the highest H → L contributions of 95% in solvent and 97% in gas, which account for the red-shift observed in the absorption spectra of these compounds.

## Transition density matrix (TDM) and exciton binding energy (E_b_)

**Figure 7:**
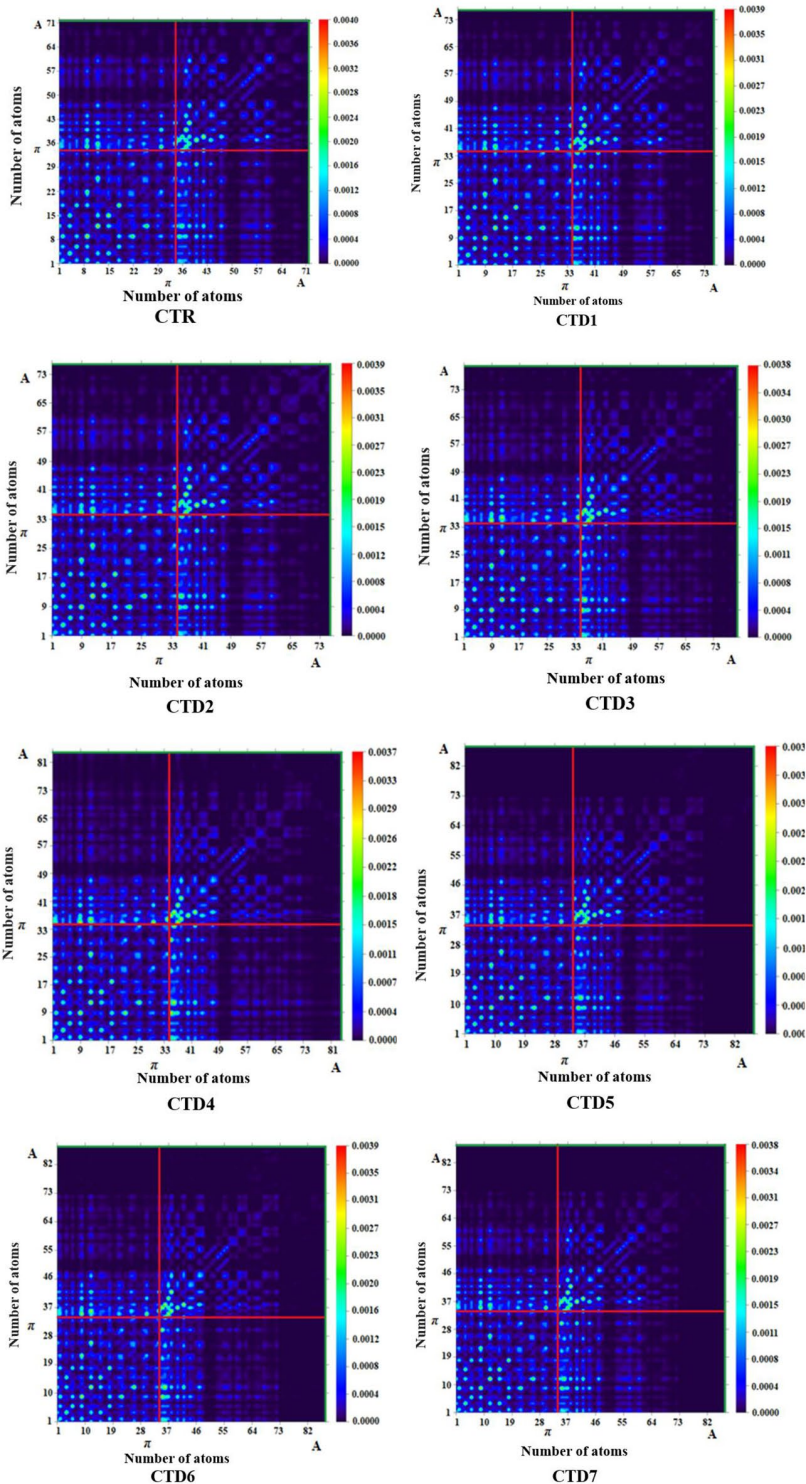
TDM graphs of designed compounds CTR and **CTD1–CTD7**.

The TDMs of designed compounds has also been examined using Multiwfn, as shown in Fig. 7. The transition density matrix provides insights into the collaboration of donor and acceptor moieties in the excited state, as well as information about location of electron–hole and electronic excitations^43^. It's noteworthy that hydrogen atoms were excluded from the analysis due to their minimal contribution in transitions.

TDM heatmaps elucidated the electronic excitation and nature of the transition in the first singlet excited state. Each molecule was divided into π-spacer (chrysene) and benzothiophene acceptor units with diverse electron-withdrawing groups (refer to Fig. 1). Almost, all the derivatives show a similar pattern of charge transference. The designed molecules (**CTD1–CTD7**) exhibited robust diagonal electron coherence in the transition density matrix (TDM) heatmaps. It is evident from the graphs that electron coherence effectively transferred from π-bridge towards the terminal acceptors (A), enabling the movement of electron density across the end-capped acceptors without entrapment. The results from TDM heat maps suggest a straightforward, smoother, and enhanced exciton dissociation in the excited state, offering potential advancements in solar cell technology.

The generation of the Frenkel exciton through the photoexcitation of small, conjugated molecules is a widely acknowledged phenomenon. In this phase, charge carriers are tightly bound by electrostatic forces, as opposed to existing as free charges. The successful dissociation of excitons leads to the creation of electrons and holes, a process made feasible by the presence of designed structures with low exciton binding energies^44^. The binding energy (*E*_*b*_) is determined by subtracting energy required for optical transition (represented by *ΔE*) and the energy gap (*E*_*g*_) between the HOMO and LUMO energy levels^45^. The equation used to calculate *E*_*b*_ is expressed as follows:

1 $${E}_{b}={E}_{g}-\Delta E$$

According to above equation, *ΔE* represents energy required by electron for ground state to the first singlet excitation, while *E*_*g*_ denotes the energy gap of HOMO and LUMO^46^. The exciton binding energy (*E*_*b*_) calculations are provided in Table 3. Notably, the *E*_*b*_ value for the designed molecule **CTD4** is the lowest (0.519 eV) among all the derivatives, making it an effective candidate to be used in new generation OSCs. The overall increasing trend of *E*_*b*_ for tailored chromophores is found as: **CTD4 < CTD6 = CTD3 < CTD5 < CTD7 = CTD1 < CTD2 < CTR**.

**Table 3:** Calculated exciton binding energies for CTR and **CTD1–CTD7** derivatives.

Compounds	*Eg*	*ΔE*	*E*_*b*_
CTR	2.493	1.904	0.589
CTD1	2.468	1.886	0.582
CTD2	2.456	1.873	0.583
CTD3	2.404	1.827	0.577
CTD4	2.327	1.808	0.519
CTD5	2.442	1.861	0.581
CTD6	2.400	1.823	0.577
CTD7	2.451	1.869	0.582

## Molecular electrostatic potential (MEP)

**Figure 8:**
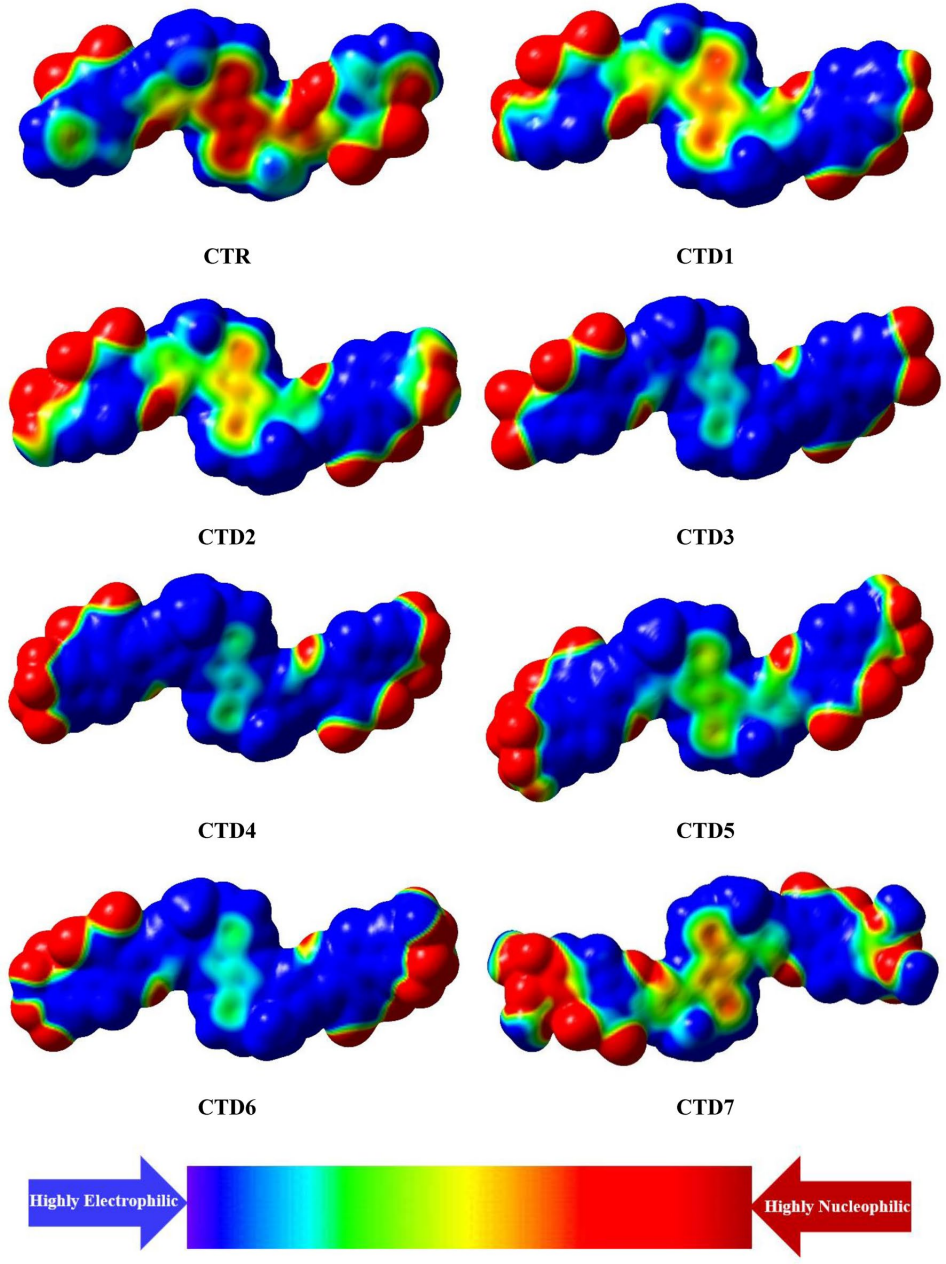
Molecular electrostatic potential diagrams of investigated molecules **CTR** and **CTD1–CTD7**.

MEPs are assessed to elucidate the quantitative representation of intermolecular charge distribution between the donor and acceptor regions of a molecular system^47^. MEP surfaces resemble a cloud with three main colors, each indicating a specific strength of the molecule's electrostatic potential. Red hues denote negative electrostatic potential, indicating electron-rich regions that could potentially serve as attack sites for electrophiles due to the intense electron density. Conversely, blue shades denote positive electrostatic potentials, signifying electron-deficient areas prone to nucleophilic attack and repulsive regions for protons due to the molecular nuclei's presence. Green-yellow shaded sites depict regions with a neutral electrostatic potential^48,49^. MEP graphs for all the studied molecules are illustrated in Fig. 8. Interestingly, a noticeable occurrence of red color is observed at the acceptor regions of all designed compounds **CTD1–CTD7** compared to **CTR** molecule, indicating them as nucleophilic region. A distinct bluish cloud is particularly prominent in the central π-region, characterizing the π-spacer as an electrophilic site. Specifically, this observation is more prominent in the **CTD3** and **CTD4** compounds. This analysis indicates increased charge separation in investigated compounds, suggesting that these newly devised molecules could be effectively synthesized for OSCs.

## Open circuit voltage (***V***_***oc***_)

Measuring open-circuit voltage (*Voc*) of the device is essential for evaluating the efficiency of an organic solar cell (OSC). The *V*_*oc*_ represents the maximum voltage generated by a solar device when applied current is 0. Various factors such as: charge transfer, temperature of photovoltaic device, and light intensity significantly influence *V*_*oc*_^50^. When the highest voltage is achieved, HOMO of donor material couples with LUMO of acceptor substance. To enhance *V*_*oc*_ values, the donor's HOMO energy state should be lower, and the acceptor's LUMO energy state should be higher^51^. In this study, the *V*_*oc*_ of designed acceptor molecules **CTR** and **CTD1–CTD7** was computed by combining these molecules with a **J52Cl** donor. According to available data, **J52Cl** is an efficient donor with HOMO/LUMO energies of − 5.299 and − 2.141 eV, respectively^52^. So, Eq. (2) has been utilized to provide a numerical estimate of *V*_*oc*_ results of all studied chromophores.

2 $${V}_{oc}=\frac{1}{e}\left(\left|{E}_{HOMO}\left(\text{Donor}\right)-{E}_{LUMO}(\text{Acceptor})\right|\right)-0.3$$

**Figure 9:**
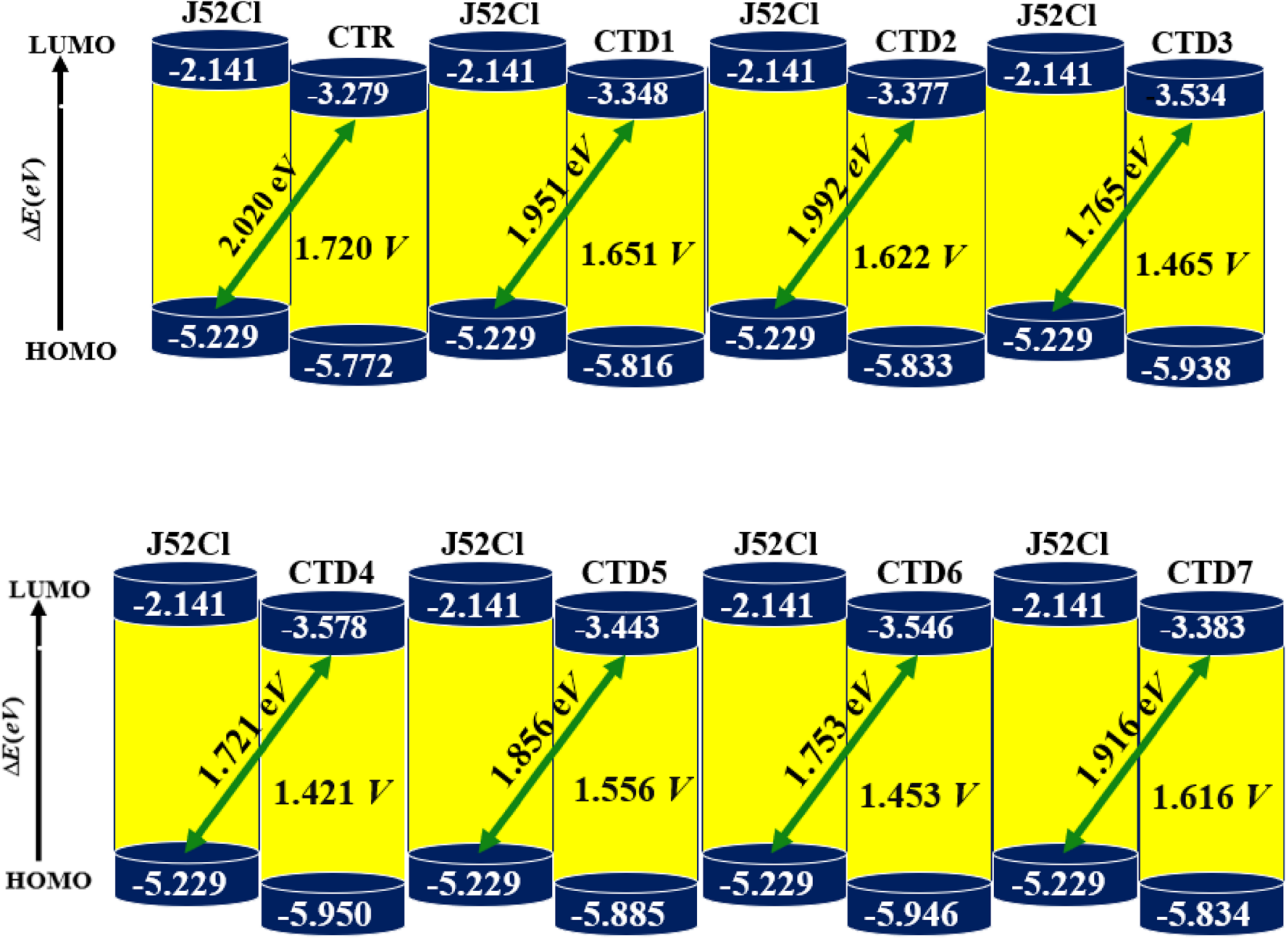
Open circuit voltage diagrams for compounds **CTR** and** CTD1–CTD7.**

Figure 9 elucidates the *V*_*oc*_ calculations achieved through blending J52Cl (donor polymer) with variously designed acceptors **CTD1–CTD7**. Amongst all the studied chromophores **CTR** and **CTD1–CTD7**, the **CTR** depicted the highest value of open circuit voltage (1.72 V). On the other hand, compound **CTD4** showed the lowest value (1.421 V) of *V*_*oc*_. Moreover, other entitled molecules represented intermediate results. Thus, the following trend in the open circuit voltage results is observed: **CTR > CTD1 > CTD2 > CTD7 > CTD5 > CTD3 > CTD6 > CTD4.** It is obvious from the results that all the derivatives exhibited maximum value of *V*_*oc*_, making them proficient OSCs material.

## Conclusion

The present research employs theoretical calculations to explore the influence of structural modifications by substituting end-capped acceptor groups in OSCs featuring the chrysene unit.

By structural modeling of chrysene unit with benzothiophene acceptors, the photovoltaic and optoelectronic properties of organic chromophores have been tuned. The quantum chemical findings demonstrated proficient charge transfer from central *π*-conjugated bridge to end-capped acceptor moieties in all the molecules. Open circuit voltage was calculated by developing a complex between designed chromophore and donor polymer (HOMOdonor–LUMOacceptor) and interestingly, higher photovoltaic response was seen in derivatives. The designed molecule **CTD4** demonstrated good properties, i.e. lowest *E*_*b*_ value (0.519 eV) and minimum band gap (2.327 eV) with maximum red-shifted absorption in both the solvent (685.791 nm) and gaseous phase (647.606 nm) amongst all **CTD1–CTD7**. Moreover, by relating the results of FMOs with GRPs, it is found that, **CTD4** derived molecule is observed as softest (0.421 eV^−1^) molecule amongst all the studied derivatives portraying its maximum charge transferring properties. Thus, **CTD4** can be considered as a remarkable material for future OSCs applications.

### Supplementary Information

Supplementary Information.

## Data Availability

All data generated or analyzed during this study are included in this published article and its supplementary information files.
