# Supplementary material for: Exploration of the synergistic effect of chrysene-based core and benzothiophene acceptors on photovoltaic properties of organic solar cells
Source: Sci Rep. 2024 Jul 2;14:15105. doi: 10.1038/s41598-024-65459-6 (PMC11219797; doi:10.1038/s41598-024-65459-6)
Supplement: Supplementary file 1 — Supplementary Information. [file 41598_2024_65459_MOESM1_ESM.docx]

**Supplementary Data**

**Exploration of the Synergistic Effect of Chrysene-based Core and Benzothiophene Acceptors on Photovoltaic properties of Organic Solar Cells**

Iqra Shafiq, ^#1,2^ Shehla Kousar,^1,2^ Faiz Rasool,^3^ Tansir Ahamad,^4^ Khurram Shahzad Munawar,^5,6^ Saifullah Bullo, *^7^ Suvash Chandra Ojha, ^#^*^8^

^1^Institute of Chemistry, Khwaja Fareed University of Engineering & Information Technology, Rahim Yar Khan, 64200, Pakistan

^2^Centre for Theoretical and Computational Research, Khwaja Fareed University of Engineering & Information Technology, Rahim Yar Khan, 64200, Pakistan

^3^Institute of Chemical Sciences, Bahauddin Zakariya University, Multan, 60800, Pakistan

^4^Department of Chemistry, King Saud University, Riyadh 11451, Saudi Arabia

^5^Institute of Chemistry, University of Sargodha, Sargodha, 40100, Pakistan

^6^Department of Chemistry, University of Mianwali, Mianwali, 42200, Pakistan

^7^Department of Human and Rehabilitation Sciences, Begum Nusrat Bhutto Women University, Sukkur Sindh Pakistan

^8^Department of Infectious Diseases, The Affiliated Hospital of Southwest Medical University, Luzhou 646000, China

^#^Both authors contribute equally

*Corresponding author's E-mail addresses:

Dr. Saifullah Bullo ([saifullah.bullo@bnbwu.edu.pk](mailto:saifullah.bullo@bnbwu.edu.pk))

Dr. Suvash Chandra Ojha ([suvash_ojha@swmu.edu.cn](mailto:suvash_ojha@swmu.edu.cn))

**Table S1:** Energies of frontier molecular orbitals of the **CTR** and **CTD1-CTD7.**

| **Compound** | **HOMO-1** | **LUMO+1** | ***Eg*** |
| --- | --- | --- | --- |
| **CTR** | -6.665 | -3.126 | 3.539 |
| **CTD1** | -6.729 | -3.203 | 3.526 |
| **CTD2** | -6.748 | -3.239 | 3.509 |
| **CTD3** | -6.885 | -3.413 | 3.472 |
| **CTD4** | -6.899 | -3.437 | 3.462 |
| **CTD5** | -6.827 | -3.306 | 3.521 |
| **CTD6** | -6.896 | -3.417 | 3.479 |
| **CTD7** | 6.759 | -3.241 | 3.528 |

Band gap = *E*_LUMO_−*E*_HOMO_, Units in *eV*

**Table S2:** Energies of frontier molecular orbitals of the **CTR** and **CTD1-CTD7.**

| **Compound** | **HOMO-2** | **LUMO+2** | ***Eg*** |
| --- | --- | --- | --- |
| **CTR** | -6.895 | -3.002 | 3.893 |
| **CTD1** | -6.937 | -3.083 | 3.854 |
| **CTD2** | -6.951 | -3.140 | 3.811 |
| **CTD3** | -7.030 | -3.389 | 3.641 |
| **CTD4** | -7.038 | -3.433 | 3.605 |
| **CTD5** | -6.990 | -3.244 | 3.746 |
| **CTD6** | -7.035 | -3.546 | 3.489 |
| **CTD7** | -6.951 | -3.137 | 3.814 |

Band gap = *E*_LUMO_−*E*_HOMO_, Units in *eV*

| **HOMO-1** | **LUMO+1** |
| --- | --- |
| 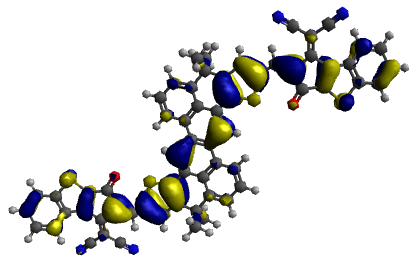 | 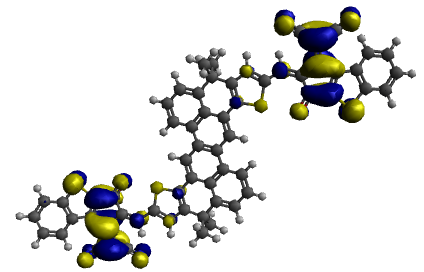 |
| **HOMO-2** | **LUMO+2** |
| 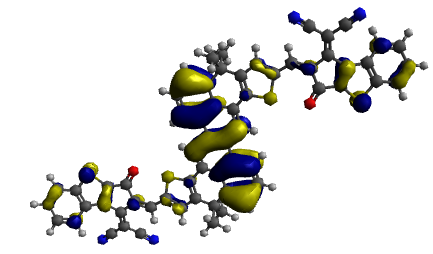 | 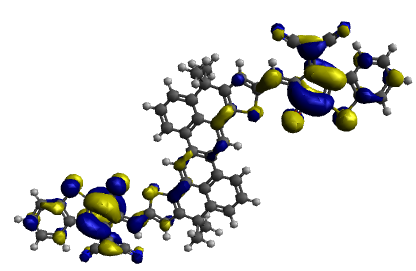 |
| **CTR** | |
| **HOMO-1** | **LUMO+1** |
| 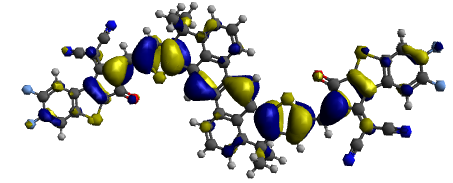 | 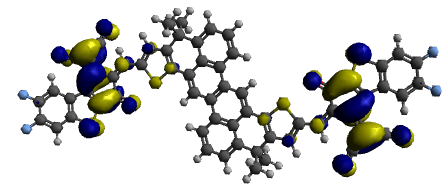 |
| **HOMO-2** | **LUMO+2** |
| 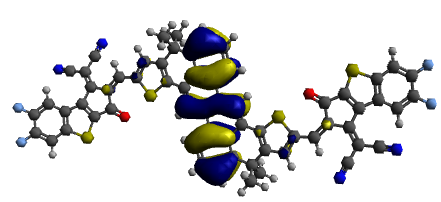 | 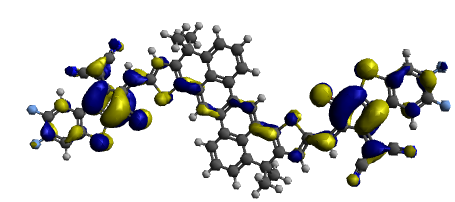 |
| **CTD1** | |
| **HOMO-1** | **LUMO+1** |
| 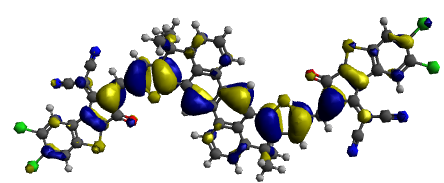 | 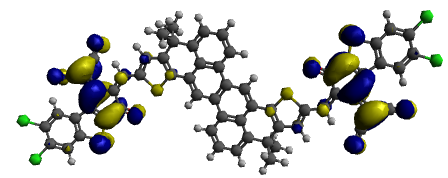 |
| **HOMO-2** | **LUMO+2** |
| 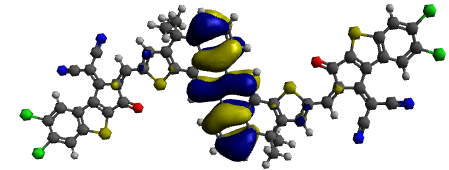 | 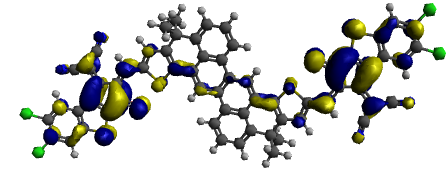 |
| **CTD2** | |
| **HOMO-1** | **LUMO+1** |
| 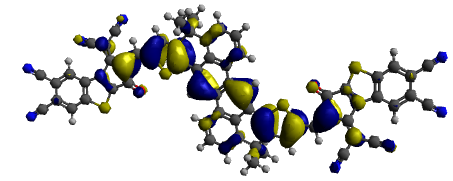 | 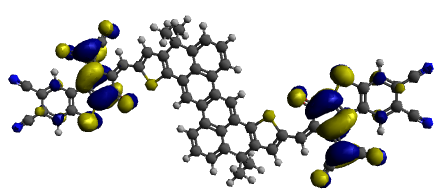 |
| **HOMO-2** | **LUMO+2** |
| 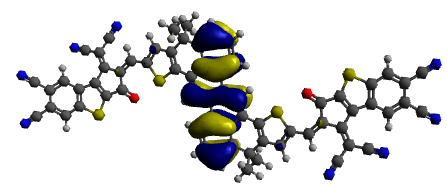 | 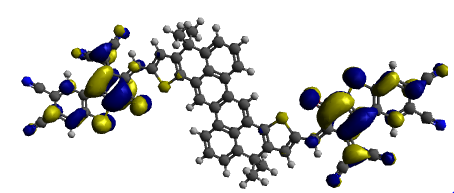 |
| **CTD3** | |
| **HOMO-1** | **LUMO+1** |
| 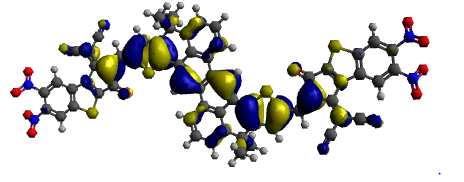 | 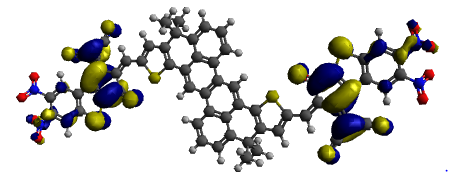 |
| **HOMO-2** | **LUMO+2** |
| 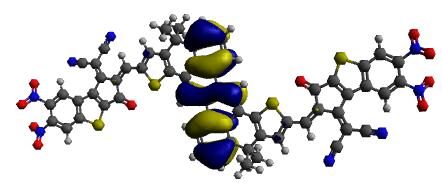 | 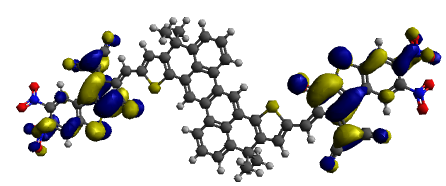 |
| **CTD4** | |
| **HOMO-1** | **LUMO+1** |
| 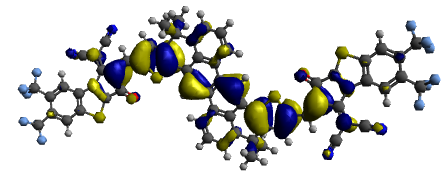 | 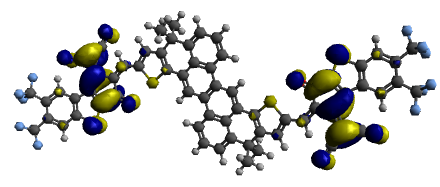 |
| **HOMO-2** | **LUMO+2** |
| 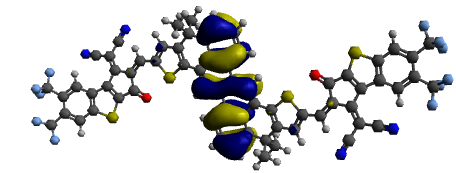 | 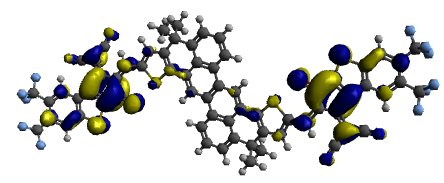 |
| **CTD5** | |
| **HOMO-1** | **LUMO+1** |
| 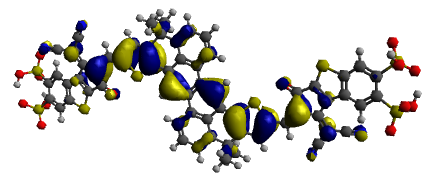 | 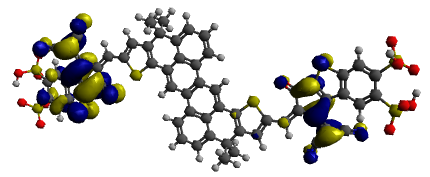 |
| **HOMO-2** | **LUMO+2** |
| 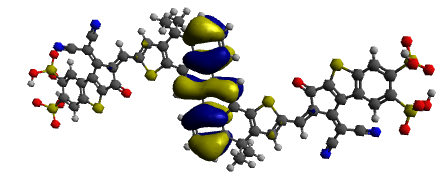 | 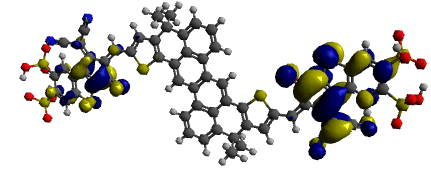 |
| **CTD6** | |
| **HOMO-1** | **LUMO+1** |
| 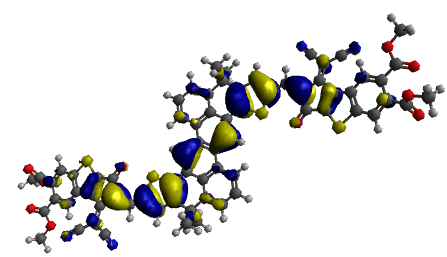 | 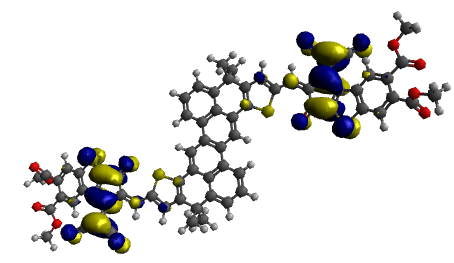 |
| **HOMO-2** | **LUMO+2** |
| 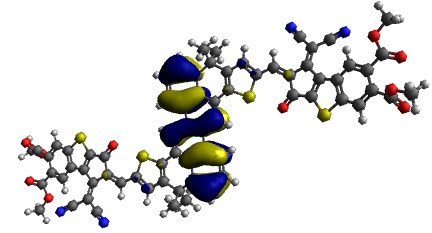 | 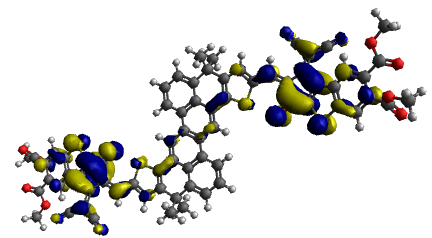 |
| **CTD7** | |

**Figure S1:** HOMO-1/LUMO+1 and HOMO-2/LUMO+2 orbital diagrams of designed compounds **CTR** and **CTD1-CTD7**

**Global Reactivity Descriptors (GRDs)**

To assess the inherent stability and chemical reactivity of designed chromophores, global reactivity descriptors were determined in this study. The reactivity and stability of a compound can be elucidated through global reactivity descriptors *i.e.,* global hardness (*η*), chemical potential (*μ*), electronegativity (*X*), global softness ($\sigma$), and electrophilicity (*ω*).^1,2^ Table S3 presents the global reactivity characteristics of the studied systems. In this context, electron affinity has been determined by taking negative values of the LUMO energy, while the ionization potential has been computed by taking negative value of the HOMO energy. Equations (1) and (2) are employed to determine the ionization potential and electron affinity of designed compounds.^3^ By utilizing Koopmans' theorem^4^, one can calculate chemical hardness (*η*), chemical potential (*μ*), electronegativity (*X*), global softness (*σ*), and electrophilicity index (*ω*) by employing Equations (3-7). The capacity of a compound to uptake additional electrical charge from its surroundings is represented as *ΔNmax*, and is determined through Equation (8).^5^

| $IP=-E_{HOMO}$ | (1) |
| --- | --- |
| $EA=-E_{LUMO}$ | (2) |
| $X=\frac{\left[ IP+EA \right]}{2}$ | (3) |
| $\eta=\frac{\left[ IP-EA \right]}{2}$ | (4) |
| $\mu=\frac{E_{HOMO}+E_{LUMO}}{2}$ | (5) |
| $\omega=\frac{\mu^{2}}{2\eta}$ | (6) |
| $\sigma=\frac{1}{2\eta}$ | (7) |
| $\Delta Nmax=\frac{\mu}{\eta}$ | (8) |

The findings of Table S3 reveal that all the compounds under investigation exhibited a high ionization potential accompanied by a low electron affinity. Notably, among the examined compounds, **CTD4** demonstrated the highest ionization potential (5.950 *eV*). The ionization potential depicted the following decreasing order: **CTD4 > CTD6 > CTD3 > CTD5 > CTD7 > CTD2 > CTD1 > CTR**. Furthermore, **CTD4** displayed highest softness value of 0.421 *eV^-1^* owing to its smallest band gap (2.327 *eV*) amongst all the derived molecules, making it the most reactive and polarizable compound. The reference compound **CTR** exhibited the highest hardness value (1.246 *eV*) indicating its maximum stability and least reactivity. Thus, softness of all the derivatives followed the following decreasing order: **CTD4 > CTD6 > CTD3 > CTD5 > CTD2 = CTD7 > CTD1 > CTR**. Additionally, **CTD4** and **CTR** exhibited the highest (4.764 *eV*) and lowest (4.525 *eV*) electronegativity values, respectively. **CTD4** displayed the largest *ΔNmax* value at 4.016 *eV*, with the decreasing order of *ΔNmax* as: **CTD4 > CTD6 > CTD3 > CTD5 > CTD7 > CTD2 > CTD1 > CTR**. In conclusion, the collective results from GRPs indicated that **CTD4** possesses the highest charge transfer properties making it the optimal choice for future OSCs applications.

**Table S3:** Ionization potential (*IP*), electron affinity (*EA*), electronegativity (*X*), chemical potential (*𝜇*), global hardness (*ƞ*), global softness (*σ*), global electrophilicity (𝜔), and charge transfer index (*ΔNmax)*.

| **Compound** | ***IP (eV)*** | ***EA (eV)*** | ***X (eV)*** | ***Ƞ (eV)*** | ***𝜇 (eV)*** | ***𝝎*** *(eV)* | ***σ (****eV^-^*^1^*)* | ***ΔNmax*** |
| --- | --- | --- | --- | --- | --- | --- | --- | --- |
| **R** | 5.772 | 3.279 | 4.525 | 1.246 | -4.525 | 8.215 | 0.401 | 3.631 |
| **D1** | 5.816 | 3.348 | 4.582 | 1.234 | -4.582 | 8.506 | 0.405 | 3.713 |
| **D2** | 5.833 | 3.377 | 4.605 | 1.228 | -4.605 | 8.634 | 0.407 | 3.750 |
| **D3** | 5.938 | 3.534 | 4.736 | 1.202 | -4.736 | 9.330 | 0.415 | 3.940 |
| **D4** | 5.950 | 3.578 | 4.764 | 1.186 | -4.764 | 9.568 | 0.421 | 4.016 |
| **D5** | 5.885 | 3.443 | 4.664 | 1.221 | -4.664 | 8.907 | 0.409 | 3.819 |
| **D6** | 5.946 | 3.546 | 4.746 | 1.200 | -4.746 | 9.385 | 0.416 | 3.955 |
| **D7** | 5.834 | 3.383 | 4.608 | 1.225 | -4.608 | 8.665 | 0.407 | 3.761 |

**Table S4:** HOMO/LUMO percentage of Density of States (DOS) of Compounds **CTR and CTD1-CTD7**

| **Compound** | **LUMO** | | **HOMO** | |
| --- | --- | --- | --- | --- |
|  | **π-spacer** | **Acceptor** | **π -spacer** | **Acceptor** |
| **CTR** | 34.9 | 65.1 | 83.5 | 16.5 |
| **CTD1** | 33.8 | 66.2 | 83.6 | 16.4 |
| **CTD2** | 34.1 | 65.9 | 83.5 | 16.4 |
| **CTD3** | 37.5 | 62.5 | 83.6 | 16.4 |
| **CTD4** | 36.5 | 63.5 | 83.5 | 16.5 |
| **CTD5** | 36.2 | 63.8 | 83.8 | 16.2 |
| **CTD6** | 38.7 | 61.3 | 83.6 | 16.4 |
| **CTD7** | 33.9 | 66.1 | 83.7 | 16.3 |

**Table S5:** Wavelength *λ*, excitation energy (*E*), oscillator strength (*f*_os_) and nature of molecular orbital contributions of compound **CTR** in gas phase

| **NO** | **DFT λ (nm)** | **E(eV)** | ***f*** | **MO contributions** |
| --- | --- | --- | --- | --- |
| 1 | 617.913 | 2.007 | 1.797 | H→L (96%), H-1→L+1 (2%) |
| 2 | 565.673 | 2.192 | 0.000 | H→L+1 (91%), H-1→L+2 (4%) |
| 3 | 549.868 | 2.255 | 0.276 | H→L+2 (92%), H-1→L+1 (3%) |
| 4 | 489.205 | 2.534 | 0.000 | H→L+3 (94%), H→L+1 (2%) |
| 5 | 427.060 | 2.903 | 0.000 | H-1→L (72%), H-5→L+1 (4%), H-3→L+1 (5%), H-2→L+1 (3%), H-1→L+2 (7%), H→L+1 (3%), H→L+3 (3%) |
| 6 | 419.986 | 2.952 | 0.059 | H-1→L+1 (59%), H-5→L (2%), H-5→L+2 (3%), H-4→L+1 (3%), H-3→L (5%), H-3→L+2 (4%), H-2→L (9%), H→L (2%), H→L+2 (5%) |

**Table S6:** Wavelength *λ*, excitation energy (*E*), oscillator strength (*f*_os_) and nature of molecular orbital contributions of compound **CTD1** in gas phase

| **NO** | **DFT λ (nm)** | **E(eV)** | ***f*** | **MO contributions** |
| --- | --- | --- | --- | --- |
| 1 | 625.204 | 1.983 | 1.747 | H→L (96%), |
| 2 | 576.242 | 2.152 | 0.000 | H→L+1 (92%), H-1→L+2 (4%) |
| 3 | 560.254 | 2.213 | 0.306 | H→L+2 (93%), H-1→L+1 (3%) |
| 4 | 495.204 | 2.504 | 0.000 | H→L+3 (95%), H→L+1 (2%) |
| 5 | 428.714 | 2.892 | 0.000 | H-1→L (74%), H-3→L+1 (9%), H-1→L+2 (6%), H→L+1 (3%), H→L+3 (3%) |
| 6 | 421.901 | 2.939 | 0.046 | H-1→L+1 (62%), H-4→L+1 (2%), H-3→L (8%), H-3→L+2 (7%), H-2→L (7%), H→L (2%), H→L+2 (4%) |

**Table S7:** Wavelength *λ*, excitation energy (*E*), oscillator strength (*f*_os_) and nature of molecular orbital contributions of compound **CTD2** in gas phase

| **NO** | **DFT λ (nm)** | **E(eV)** | ***f*** | **MO contributions** |
| --- | --- | --- | --- | --- |
| 1 | 628.627 | 1.972 | 1.882 | H→L (96%), |
| 2 | 579.420 | 2.140 | 0.000 | H→L+1 (92%), H-1→L+2 (4%) |
| 3 | 566.837 | 2.187 | 0.279 | H→L+2 (93%), H-1→L+1 (3%) |
| 4 | 500.320 | 2.478 | 0.000 | H→L+3 (95%), |
| 5 | 430.471 | 2.880 | 0.000 | H-1→L (70%), H-5→L+1 (3%), H-3→L+1 (8%), H-1→L+2 (9%), H→L+1 (3%), H→L+3 (2%) |
| 6 | 424.284 | 2.922 | 0.033 | H-1→L+1 (62%), H-5→L+2 (3%), H-4→L+1 (3%), H-3→L (7%), H-3→L+2 (6%), H-2→L (6%), H→L+2 (5%) |

**Table S8:** Wavelength *λ*, excitation energy (*E*), oscillator strength (*f*_os_) and nature of molecular orbital contributions of compound **CTD3** in gas phase

| **NO** | **DFT λ (nm)** | **E(eV)** | ***f*** | **MO contributions** |
| --- | --- | --- | --- | --- |
| 1 | 643.840 | 1.926 | 2.031 | H→L (97%), |
| 2 | 602.508 | 2.058 | 0.000 | H→L+1 (94%), H-1→L+2 (4%) |
| 3 | 599.653 | 2.068 | 0.101 | H→L+2 (94%), H-1→L+1 (3%) |
| 4 | 524.890 | 2.362 | 0.000 | H→L+3 (98%), |
| 5 | 432.106 | 2.869 | 0.000 | H-1→L (60%), H-1→L+2 (21%), H-3→L+1 (8%), H→L+1 (3%) |
| 6 | 428.433 | 2.894 | 0.018 | H-3→L+2 (10%), H-1→L+1 (67%), H-6→L+1 (2%), H-3→L (2%), H-2→L (6%), H→L+2 (5%) |

**Table S9:** Wavelength *λ*, excitation energy (*E*), oscillator strength (*f*_os_) and nature of molecular orbital contributions of compound **CTD4** in gas phase

| **NO** | **DFT λ (nm)** | **E(eV)** | ***f*** | **MO contributions** |
| --- | --- | --- | --- | --- |
| 1 | 647.606 | 1.915 | 2.027 | H→L (97%), |
| 2 | 602.216 | 2.059 | 0.000 | H→L+1 (94%), H-1→L+2 (3%) |
| 3 | 600.728 | 2.064 | 0.050 | H→L+2 (94%), H-1→L+1 (3%) |
| 4 | 533.105 | 2.326 | 0.000 | H→L+3 (98%), |
| 5 | 435.063 | 2.850 | 0.089 | H→L+4 (87%), H-1→L+1 (4%) |
| 6 | 432.543 | 2.866 | 0.000 | H-1→L (65%), H-1→L+2 (16%), H-3→L+1 (5%), H→L+1 (2%), H→L+5 (5%) |

**Table S10:** Wavelength *λ*, excitation energy (*E*), oscillator strength (*f*_os_) and nature of molecular orbital contributions of compound **CTD5** in gas phase

| **NO** | **DFT λ (nm)** | **E(eV)** | ***f*** | **MO contributions** |
| --- | --- | --- | --- | --- |
| 1 | 632.863 | 1.959 | 1.901 | H→L (96%), |
| 2 | 586.020 | 2.116 | 0.000 | H→L+1 (93%), H-1→L+2 (4%) |
| 3 | 579.203 | 2.141 | 0.162 | H→L+2 (94%), H-1→L+1 (3%) |
| 4 | 510.938 | 2.427 | 0.001 | H→L+3 (97%), |
| 5 | 427.561 | 2.900 | 0.001 | H-1→L (74%), H-3→L+1 (7%), H-1→L+2 (9%), H→L+1 (3%) |
| 6 | 421.672 | 2.940 | 0.049 | H-2→L (13%), H-1→L+1 (61%), H-6→L+1 (3%), H-3→L (4%), H-3→L+2 (8%), H→L+2 (4%) |

**Table S11:** Wavelength *λ*, excitation energy (*E*), oscillator strength (*f*_os_) and nature of molecular orbital contributions of compound **CTD6** in gas phase

| **NO** | **DFT λ (nm)** | **E(eV)** | ***f*** | **MO contributions** |
| --- | --- | --- | --- | --- |
| 1 | 644.643 | 1.923 | 2.092 | H→L (97%), |
| 2 | 603.976 | 2.053 | 0.027 | H→L+1 (95%), H-1→L+1 (3%) |
| 3 | 594.734 | 2.085 | 0.009 | H→L+2 (94%), H-1→L+2 (4%) |
| 4 | 527.189 | 2.352 | 0.003 | H→L+3 (98%), |
| 5 | 429.978 | 2.884 | 0.003 | H-1→L (63%), H-1→L+1 (21%), H-3→L+1 (5%), H→L+1 (2%) |
| 6 | 425.434 | 2.914 | 0.040 | H-2→L (25%), H-1→L (19%), H-1→L+1 (33%), H-3→L+1 (3%), H-1→L+2 (7%) |

**Table S12:** Wavelength *λ*, excitation energy (*E*), oscillator strength (*f*_os_) and nature of molecular orbital contributions of compound **CTD7** in gas phase

| **NO** | **DFT λ (nm)** | **E(eV)** | ***f*** | **MO contributions** |
| --- | --- | --- | --- | --- |
| 1 | 628.819 | 1.972 | 1.915 | H→L (96%), |
| 2 | 575.573 | 2.154 | 0.001 | H→L+1 (92%), H-1→L+2 (4%) |
| 3 | 561.014 | 2.210 | 0.278 | H→L+2 (93%), H-1→L+1 (3%) |
| 4 | 498.409 | 2.488 | 0.001 | H→L+3 (94%), H→L+1 (2%) |
| 5 | 429.769 | 2.885 | 0.001 | H-1→L (74%), H-3→L+1 (7%), H-1→L+2 (6%), H→L+1 (3%), H→L+3 (2%) |
| 6 | 422.433 | 2.935 | 0.058 | H-1→L+1 (60%), H-4→L+1 (3%), H-3→L (8%), H-3→L+2 (6%), H-2→L (7%), H→L (2%), H→L+2 (5%) |

**Table S13:** Wavelength *λ*, excitation energy (*E*), oscillator strength (*f*_os_) and nature of molecular orbital contributions of compound **CTR** in solvent phase

| **NO** | **DFT λ (nm)** | **E(eV)** | ***f*** | **MO contributions** |
| --- | --- | --- | --- | --- |
| 1 | 651.109 | 1.904 | 2.005 | H→L (94%), H-1→L+1 (2%) |
| 2 | 583.263 | 2.126 | 0.000 | H→L+1 (92%), H-1→L (2%), H-1→L+2 (3%) |
| 3 | 561.701 | 2.207 | 0.273 | H→L+2 (90%), H-1→L+1 (4%) |
| 4 | 501.027 | 2.475 | 0.000 | H→L+3 (96%), |
| 5 | 441.586 | 2.808 | 0.000 | H-1→L (78%), H-5→L+1 (2%), H-3→L+1 (5%), H-2→L+1 (5%), H→L+1 (5%) |
| 6 | 431.339 | 2.874 | 0.106 | H-2→L (12%), H-1→L+1 (57%), H-5→L (2%), H-4→L+1 (3%), H-3→L (7%), H-3→L+2 (4%), H-2→L+2 (2%), H→L (3%), H→L+2 (5%) |

**Table S14:** Wavelength *λ*, excitation energy (*E*), oscillator strength (*f*_os_) and nature of molecular orbital contributions of compound **CTD1** in solvent phase

| **NO** | **DFT λ (nm)** | **E(eV)** | ***f*** | **MO contributions** |
| --- | --- | --- | --- | --- |
| 1 | 657.497 | 1.886 | 1.979 | H→L (94%), H-1→L+1 (2%), H→L+2 (2%) |
| 2 | 591.048 | 2.098 | 0.000 | H→L+1 (92%), H-1→L (2%), H-1→L+2 (3%) |
| 3 | 569.937 | 2.175 | 0.278 | H→L+2 (91%), H-1→L+1 (3%) |
| 4 | 506.513 | 2.448 | 0.000 | H→L+3 (96%), |
| 5 | 441.696 | 2.807 | 0.000 | H-1→L (81%), H-3→L+1 (8%), H→L+1 (4%) |
| 6 | 430.860 | 2.878 | 0.070 | H-3→L (11%), H-1→L+1 (61%), H-4→L+1 (2%), H-3→L+2 (6%), H-2→L (7%), H→L (3%), H→L+2 (4%) |

**Table S15:** Wavelength *λ*, excitation energy (*E*), oscillator strength (*f*_os_) and nature of molecular orbital contributions of compound **CTD2** in solvent phase

| **NO** | **DFT λ (nm)** | **E(eV)** | ***f*** | **MO contributions** |
| --- | --- | --- | --- | --- |
| 1 | 662.061 | 1.873 | 2.081 | H→L (93%), H→L+2 (2%) |
| 2 | 595.992 | 2.080 | 0.000 | H→L+1 (92%), H-1→L (2%), H-1→L+2 (3%) |
| 3 | 578.716 | 2.142 | 0.261 | H→L+2 (91%), H-1→L+1 (4%) |
| 4 | 513.626 | 2.414 | 0.000 | H→L+3 (97%), |
| 5 | 443.339 | 2.797 | 0.000 | H-1→L (80%), H-3→L+1 (8%), H→L+1 (4%) |
| 6 | 433.360 | 2.861 | 0.058 | H-3→L (10%), H-1→L+1 (61%), H-4→L+1 (3%), H-3→L+2 (6%), H-2→L (7%), H→L (3%), H→L+2 (4%) |

**Table S16:** Wavelength *λ*, excitation energy (*E*), oscillator strength (*f*_os_) and nature of molecular orbital contributions of compound **CTD3** in solvent phase

| **NO** | **DFT λ (nm)** | **E(eV)** | ***f*** | **MO contributions** |
| --- | --- | --- | --- | --- |
| 1 | 678.510 | 1.827 | 2.228 | H→L (95%), |
| 2 | 613.601 | 2.021 | 0.000 | H→L+1 (94%), H-1→L+2 (3%) |
| 3 | 608.571 | 2.037 | 0.096 | H→L+2 (93%), H-1→L+1 (4%) |
| 4 | 542.126 | 2.287 | 0.000 | H→L+3 (98%), |
| 5 | 443.799 | 2.794 | 0.000 | H-1→L (84%), H-3→L+1 (5%), H-1→L+2 (3%), H→L+1 (2%) |
| 6 | 434.575 | 2.853 | 0.034 | H-1→L+1 (64%), H-4→L+1 (2%), H-3→L (5%), H-3→L+2 (9%), H-2→L (8%), H→L+2 (4%) |

**Table S17:** Wavelength *λ*, excitation energy (*E*), oscillator strength (*f*_os_) and nature of molecular orbital contributions of compound **CTD4** in solvent phase

| **NO** | **DFT λ (nm)** | **E(eV)** | ***f*** | **MO contributions** |
| --- | --- | --- | --- | --- |
| 1 | 685.791 | 1.808 | 2.210 | H→L (95%), |
| 2 | 615.398 | 2.015 | 0.000 | H→L+1 (94%), H-1→L+2 (4%) |
| 3 | 612.570 | 2.024 | 0.052 | H→L+2 (93%), H-1→L+1 (4%) |
| 4 | 559.117 | 2.218 | 0.000 | H→L+3 (95%), H→L+5 (3%) |
| 5 | 475.874 | 2.605 | 0.085 | H→L+4 (94%), |
| 6 | 459.048 | 2.701 | 0.000 | H→L+5 (93%), H→L+3 (3%) |

**Table S18:** Wavelength *λ*, excitation energy (*E*), oscillator strength (*f*_os_) and nature of molecular orbital contributions of compound **CTD5** in solvent phase

| **NO** | **DFT λ (nm)** | **E(eV)** | ***f*** | **MO contributions** |
| --- | --- | --- | --- | --- |
| 1 | 666.295 | 1.861 | 2.094 | H→L (94%), H→L+2 (2%) |
| 2 | 599.189 | 2.069 | 0.000 | H→L+1 (93%), H-1→L+2 (3%) |
| 3 | 588.021 | 2.109 | 0.160 | H→L+2 (92%), H-1→L+1 (3%) |
| 4 | 524.091 | 2.366 | 0.001 | H→L+3 (97%), |
| 5 | 440.066 | 2.817 | 0.001 | H-1→L (85%), H-3→L+1 (6%), H→L+1 (3%) |
| 6 | 429.041 | 2.890 | 0.069 | H-2→L (11%), H-1→L+1 (61%), H-4→L+1 (3%), H-3→L (8%), H-3→L+2 (7%), H→L (2%), H→L+2 (4%) |

**Table S19:** Wavelength *λ*, excitation energy (*E*), oscillator strength (*f*_os_) and nature of molecular orbital contributions of compound **CTD6** in solvent phase

| **NO** | **DFT λ (nm)** | **E(eV)** | ***f*** | **MO contributions** |
| --- | --- | --- | --- | --- |
| 1 | 680.223 | 1.823 | 2.243 | H→L (95%), |
| 2 | 612.086 | 2.026 | 0.018 | H→L+1 (93%), |
| 3 | 606.309 | 2.045 | 0.052 | H→L+2 (93%), |
| 4 | 543.576 | 2.281 | 0.001 | H→L+3 (98%), |
| 5 | 443.086 | 2.798 | 0.001 | H-1→L (88%), H-3→L+1 (3%) |
| 6 | 432.196 | 2.869 | 0.069 | H-2→L (20%), H-1→L+1 (54%), H-3→L (3%), H-3→L+2 (6%), H-1→L+2 (3%), H→L+2 (3%) |

**Table S20:** Wavelength *λ*, excitation energy (*E*), oscillator strength (*f*_os_) and nature of molecular orbital contributions of compound **CTD7** in solvent phase

| **NO** | **DFT λ (nm)** | **E(eV)** | ***f*** | **MO contributions** |
| --- | --- | --- | --- | --- |
| 1 | 663.301 | 1.869 | 2.086 | H→L (94%), H→L+2 (2%) |
| 2 | 595.791 | 2.081 | 0.001 | H→L+1 (93%), H-1→L+2 (3%) |
| 3 | 577.342 | 2.148 | 0.257 | H→L+2 (91%), H-1→L+1 (3%) |
| 4 | 513.882 | 2.413 | 0.001 | H→L+3 (97%), |
| 5 | 442.516 | 2.802 | 0.001 | H-1→L (81%), H-3→L+1 (8%), H→L+1 (4%) |
| 6 | 431.565 | 2.873 | 0.080 | H-3→L (11%), H-1→L+1 (61%), H-4→L+1 (3%), H-3→L+2 (6%), H-2→L (8%), H→L (3%), H→L+2 (4%) |

**Table S21:** ChemDraw Structures of designed compounds **CTR** and **CTD1-CTD7**

| **CTR** | **** |
| --- | --- |
|  | 2,2'-((2Z,2'Z)-2,2'-((4,4,12,12-tetramethyl-4,12-dihydrodibenzo[4,5:10,11]tetraceno[1,2-b:7,8-b']dithiophene-2,10-diyl)bis(methanylylidene))bis(3-oxo-2,3-dihydro-1H-benzo[b]cyclopenta[d]thiophene-2,1-diylidene))dimalononitrile |
| **CTD1** | **** |
|  | 2,2'-((2Z,2'Z)-2,2'-((4,4,12,12-tetramethyl-4,12-dihydrodibenzo[4,5:10,11]tetraceno[1,2-b:7,8-b']dithiophene-2,10-diyl)bis(methanylylidene))bis(6,7-difluoro-3-oxo-2,3-dihydro-1H-benzo[b]cyclopenta[d]thiophene-2,1-diylidene))dimalononitrile |
| **CTD2** | **** |
|  | 2,2'-((2Z,2'Z)-2,2'-((4,4,12,12-tetramethyl-4,12-dihydrodibenzo[4,5:10,11]tetraceno[1,2-b:7,8-b']dithiophene-2,10-diyl)bis(methanylylidene))bis(6,7-dichloro-3-oxo-2,3-dihydro-1H-benzo[b]cyclopenta[d]thiophene-2,1-diylidene))dimalononitrile |
| **CTD3** | **** |
|  | (2Z,2'Z)-2,2'-((4,4,12,12-tetramethyl-4,12-dihydrodibenzo[4,5:10,11]tetraceno[1,2-b:7,8-b']dithiophene-2,10-diyl)bis(methanylylidene))bis(1-(dicyanomethylene)-3-oxo-2,3-dihydro-1H-benzo[b]cyclopenta[d]thiophene-6,7-dicarbonitrile) |
| **CTD4** | **** |
|  | 2,2'-((2Z,2'Z)-2,2'-((4,4,12,12-tetramethyl-4,12-dihydrodibenzo[4,5:10,11]tetraceno[1,2-b:7,8-b']dithiophene-2,10-diyl)bis(methanylylidene))bis(6,7-dinitro-3-oxo-2,3-dihydro-1H-benzo[b]cyclopenta[d]thiophene-2,1-diylidene))dimalononitrile |
| **CTD5** | **** |
|  | 2,2'-((2Z,2'Z)-2,2'-((4,4,12,12-tetramethyl-4,12-dihydrodibenzo[4,5:10,11]tetraceno[1,2-b:7,8-b']dithiophene-2,10-diyl)bis(methanylylidene))bis(3-oxo-6,7-bis(trifluoromethyl)-2,3-dihydro-1H-benzo[b]cyclopenta[d]thiophene-2,1-diylidene))dimalononitrile |
| **CTD6** | **** |
|  | (2Z,2'Z)-2,2'-((4,4,12,12-tetramethyl-4,12-dihydrodibenzo[4,5:10,11]tetraceno[1,2-b:7,8-b']dithiophene-2,10-diyl)bis(methanylylidene))bis(1-(dicyanomethylene)-3-oxo-2,3-dihydro-1H-benzo[b]cyclopenta[d]thiophene-6,7-disulfonic acid) |
| **CTD7** | **** |
|  | (2Z,2'Z)-tetramethyl 2,2'-((4,4,12,12-tetramethyl-4,12-dihydrodibenzo[4,5:10,11]tetraceno[1,2-b:7,8-b']dithiophene-2,10-diyl)bis(methanylylidene))bis(1-(dicyanomethylene)-3-oxo-2,3-dihydro-1H-benzo[b]cyclopenta[d]thiophene-6,7-dicarboxylate) |

**Table S22:** Cartesian coordinates **CTR**

| **Atom** | **X-axis** | **Y-axis** | **Z-axis** |
| --- | --- | --- | --- |
| C | 0.43461 | -0.45837 | -0.10126 |
| C | -0.57682 | 0.4921 | -0.10428 |
| C | 0.03616 | -1.93725 | -0.04388 |
| C | -1.92761 | 0.09863 | -0.11927 |
| C | -0.17197 | 1.96816 | -0.05983 |
| C | 1.79081 | -0.07164 | -0.12016 |
| C | -1.31913 | -2.25896 | -0.03318 |
| C | 1.00027 | -2.9541 | 0.02429 |
| C | -2.28477 | -1.22209 | -0.04594 |
| H | -2.68866 | 0.84929 | -0.17681 |
| C | 1.19488 | 2.28258 | -0.04082 |
| C | -1.13714 | 2.99151 | -0.00983 |
| C | 2.16613 | 1.2443 | -0.05267 |
| H | 2.53832 | -0.8274 | -0.17359 |
| C | -1.69966 | -3.61681 | 0.02178 |
| C | 0.60758 | -4.24475 | 0.18623 |
| H | 2.04158 | -2.71487 | -0.04076 |
| C | -3.63532 | -1.55937 | 0.06456 |
| C | 1.57353 | 3.64082 | 0.01753 |
| C | -0.73959 | 4.28276 | 0.13483 |
| H | -2.18106 | 2.75701 | -0.07548 |
| C | 3.52818 | 1.58274 | 0.04466 |
| C | -3.1714 | -4.04008 | -0.15666 |
| C | -0.75818 | -4.57456 | 0.21065 |
| H | 1.34202 | -5.01614 | 0.29323 |
| C | -4.09522 | -2.83495 | 0.02629 |
| S | -4.95762 | -0.41191 | 0.31271 |
| C | 3.04932 | 4.05806 | -0.10974 |
| C | 0.62892 | 4.60478 | 0.17064 |
| H | -1.47106 | 5.06375 | 0.221 |
| C | 3.95688 | 2.86848 | 0.14633 |
| S | 4.88501 | 0.457 | 0.11355 |
| C | -3.35022 | -4.56961 | -1.5885 |
| C | -3.5374 | -5.14434 | 0.85476 |
| H | -1.05765 | -5.58944 | 0.36382 |
| C | -5.50344 | -2.94815 | 0.12736 |
| C | -6.14432 | -1.70137 | 0.12438 |
| C | 3.3051 | 4.55489 | -1.54037 |
| C | 3.38761 | 5.17734 | 0.87978 |
| H | 0.92909 | 5.62422 | 0.30718 |
| C | 5.31092 | 3.01219 | 0.48465 |
| C | 5.96051 | 1.82832 | 0.40114 |
| H | -4.36945 | -4.86619 | -1.73051 |
| H | -3.10039 | -3.80331 | -2.28923 |
| H | -2.70953 | -5.41385 | -1.73746 |
| H | -2.89803 | -5.989 | 0.71101 |
| H | -3.41128 | -4.76761 | 1.85088 |
| H | -4.55545 | -5.43792 | 0.71234 |
| H | -6.02235 | -3.88192 | 0.17324 |
| C | -7.51968 | -1.50414 | -0.03293 |
| H | 4.3356 | 4.82446 | -1.63069 |
| H | 3.0746 | 3.78138 | -2.24258 |
| H | 2.69155 | 5.40893 | -1.74044 |
| H | 2.77503 | 6.03229 | 0.68674 |
| H | 3.21511 | 4.83344 | 1.87766 |
| H | 4.41728 | 5.44058 | 0.7629 |
| H | 5.77927 | 3.9545 | 0.74618 |
| C | 7.45717 | 1.72572 | 0.48771 |
| C | -8.05183 | -0.29514 | 0.24245 |
| H | -8.14636 | -2.30408 | -0.37125 |
| C | 8.10384 | 0.5761 | 0.23 |
| H | 8.00865 | 2.59014 | 0.74304 |
| C | -7.36977 | 0.84041 | 1.01598 |
| C | -9.45832 | 0.21269 | -0.13534 |
| C | 7.52925 | -0.82251 | -0.06778 |
| C | 9.6146 | 0.43277 | 0.16272 |
| O | -6.20458 | 0.85043 | 1.47788 |
| C | -10.4007 | -0.53429 | -0.77357 |
| C | -9.50053 | 1.67921 | 0.37852 |
| O | 6.3218 | -1.12052 | -0.12222 |
| C | 10.53435 | 1.41724 | 0.28047 |
| C | 9.88063 | -1.0475 | -0.10536 |
| C | -11.6686 | -0.0341 | -1.08604 |
| C | -10.0768 | -1.86174 | -1.10959 |
| C | 11.88906 | 1.08509 | 0.27894 |
| C | 10.14293 | 2.76097 | 0.38445 |
| N | -12.7141 | 0.33764 | -1.36077 |
| N | -9.79112 | -2.94397 | -1.37407 |
| N | 12.99922 | 0.79806 | 0.27602 |
| N | 9.82359 | 3.85721 | 0.46928 |
| C | -8.42324 | 1.94284 | 1.11805 |
| S | -8.54342 | 3.46782 | 1.98973 |
| C | -10.4335 | 2.91281 | 0.25115 |
| C | 8.76029 | -1.73887 | -0.27054 |
| S | 9.07858 | -3.43631 | -0.66865 |
| C | 10.84534 | -3.08779 | -0.72714 |
| C | -10.0164 | 3.91065 | 1.08511 |
| C | -10.6896 | 5.12174 | 1.18463 |
| C | -11.4997 | 3.07339 | -0.56852 |
| C | -11.7905 | 5.3293 | 0.3459 |
| H | -10.3747 | 5.87036 | 1.88424 |
| C | -12.1834 | 4.31157 | -0.55263 |
| H | -11.8112 | 2.2878 | -1.22496 |
| H | -12.3278 | 6.25371 | 0.3872 |
| H | -13 | 4.48184 | -1.22345 |
| C | 11.14683 | -1.85794 | -0.25948 |
| C | 12.44437 | -1.42492 | 0.00502 |
| C | 13.48651 | -2.27282 | -0.36561 |
| H | 12.63171 | -0.47553 | 0.47259 |
| C | 11.84284 | -3.91035 | -1.18709 |
| C | 13.18153 | -3.4978 | -1.01022 |
| H | 14.50139 | -1.997 | -0.17006 |
| H | 11.59982 | -4.8289 | -1.66113 |
| H | 13.97062 | -4.11453 | -1.36226 |

**Table S23:** Cartesian coordinates **CTD1**

| **Atom** | **X-axis** | **Y-axis** | **Z-axis** |
| --- | --- | --- | --- |
| C | 0.43461 | -0.45837 | -0.10126 |
| C | -0.57682 | 0.4921 | -0.10428 |
| C | 0.03616 | -1.93725 | -0.04388 |
| C | -1.92761 | 0.09863 | -0.11927 |
| C | -0.17197 | 1.96816 | -0.05983 |
| C | 1.79081 | -0.07164 | -0.12016 |
| C | -1.31913 | -2.25896 | -0.03318 |
| C | 1.00027 | -2.9541 | 0.02429 |
| C | -2.28477 | -1.22209 | -0.04594 |
| H | -2.68866 | 0.84929 | -0.17681 |
| C | 1.19488 | 2.28258 | -0.04082 |
| C | -1.13714 | 2.99151 | -0.00983 |
| C | 2.16613 | 1.2443 | -0.05267 |
| H | 2.53832 | -0.8274 | -0.17359 |
| C | -1.69966 | -3.61681 | 0.02178 |
| C | 0.60758 | -4.24475 | 0.18623 |
| H | 2.04158 | -2.71487 | -0.04076 |
| C | -3.63532 | -1.55937 | 0.06456 |
| C | 1.57353 | 3.64082 | 0.01753 |
| C | -0.73959 | 4.28276 | 0.13483 |
| H | -2.18106 | 2.75701 | -0.07548 |
| C | 3.52818 | 1.58274 | 0.04466 |
| C | -3.1714 | -4.04008 | -0.15666 |
| C | -0.75818 | -4.57456 | 0.21065 |
| H | 1.34202 | -5.01614 | 0.29323 |
| C | -4.09522 | -2.83495 | 0.02629 |
| S | -4.95762 | -0.41191 | 0.31271 |
| C | 3.04932 | 4.05806 | -0.10974 |
| C | 0.62892 | 4.60478 | 0.17064 |
| H | -1.47106 | 5.06375 | 0.221 |
| C | 3.95688 | 2.86848 | 0.14633 |
| S | 4.88501 | 0.457 | 0.11355 |
| C | -3.35022 | -4.56961 | -1.5885 |
| C | -3.5374 | -5.14434 | 0.85476 |
| H | -1.05765 | -5.58944 | 0.36382 |
| C | -5.50344 | -2.94815 | 0.12736 |
| C | -6.14432 | -1.70137 | 0.12438 |
| C | 3.3051 | 4.55489 | -1.54037 |
| C | 3.38761 | 5.17734 | 0.87978 |
| H | 0.92909 | 5.62422 | 0.30718 |
| C | 5.31092 | 3.01219 | 0.48465 |
| C | 5.96051 | 1.82832 | 0.40114 |
| H | -4.36945 | -4.86619 | -1.73051 |
| H | -3.10039 | -3.80331 | -2.28923 |
| H | -2.70953 | -5.41385 | -1.73746 |
| H | -2.89803 | -5.989 | 0.71101 |
| H | -3.41128 | -4.76761 | 1.85088 |
| H | -4.55545 | -5.43792 | 0.71234 |
| H | -6.02235 | -3.88192 | 0.17324 |
| C | -7.51968 | -1.50414 | -0.03293 |
| H | 4.3356 | 4.82446 | -1.63069 |
| H | 3.0746 | 3.78138 | -2.24258 |
| H | 2.69155 | 5.40893 | -1.74044 |
| H | 2.77503 | 6.03229 | 0.68674 |
| H | 3.21511 | 4.83344 | 1.87766 |
| H | 4.41728 | 5.44058 | 0.7629 |
| H | 5.77926 | 3.9545 | 0.74618 |
| C | 7.45717 | 1.72572 | 0.48771 |
| C | -8.05183 | -0.29514 | 0.24245 |
| H | -8.14636 | -2.30408 | -0.37125 |
| C | 8.10384 | 0.5761 | 0.23 |
| H | 8.00865 | 2.59014 | 0.74304 |
| C | -7.36977 | 0.84041 | 1.01598 |
| C | -9.45832 | 0.21269 | -0.13534 |
| C | 7.52925 | -0.82251 | -0.06778 |
| C | 9.6146 | 0.43277 | 0.16272 |
| O | -6.20458 | 0.85043 | 1.47788 |
| C | -10.4007 | -0.53429 | -0.77357 |
| C | -9.50053 | 1.67921 | 0.37852 |
| O | 6.3218 | -1.12052 | -0.12222 |
| C | 10.53435 | 1.41724 | 0.28047 |
| C | 9.88063 | -1.0475 | -0.10536 |
| C | -11.6686 | -0.03409 | -1.08604 |
| C | -10.0768 | -1.86175 | -1.10958 |
| C | 11.88906 | 1.08509 | 0.27893 |
| C | 10.14293 | 2.76097 | 0.38445 |
| N | -12.7141 | 0.33764 | -1.36077 |
| N | -9.79112 | -2.94396 | -1.37408 |
| N | 12.99922 | 0.79806 | 0.27603 |
| N | 9.82359 | 3.85721 | 0.46928 |
| C | -8.42324 | 1.94284 | 1.11805 |
| S | -8.54342 | 3.46782 | 1.98973 |
| C | -10.4335 | 2.91281 | 0.25115 |
| C | 8.76029 | -1.73887 | -0.27054 |
| S | 9.07858 | -3.43631 | -0.66865 |
| C | 10.84535 | -3.08779 | -0.72714 |
| C | -10.0164 | 3.91065 | 1.08511 |
| C | -10.6896 | 5.12174 | 1.18463 |
| C | -11.4997 | 3.07339 | -0.56852 |
| C | -11.7905 | 5.3293 | 0.3459 |
| H | -10.3747 | 5.87036 | 1.88424 |
| C | -12.1834 | 4.31157 | -0.55263 |
| H | -11.8112 | 2.2878 | -1.22496 |
| C | 11.14683 | -1.85794 | -0.25948 |
| C | 12.44437 | -1.42492 | 0.00502 |
| C | 13.48651 | -2.27282 | -0.36561 |
| H | 12.63171 | -0.47553 | 0.47259 |
| C | 11.84284 | -3.91034 | -1.18709 |
| C | 13.18153 | -3.4978 | -1.01022 |
| H | 11.59982 | -4.8289 | -1.66113 |
| F | -13.2133 | 4.52631 | -1.39866 |
| F | -12.4684 | 6.49558 | 0.39801 |
| F | 14.76731 | -1.92472 | -0.11882 |
| F | 14.18501 | -4.28209 | -1.4579 |

**Table S24:** Cartesian coordinates **CTD2**

| **Atom** | **X-axis** | **Y-axis** | **Z-axis** |
| --- | --- | --- | --- |
| C | 0.43461 | -0.45837 | -0.10126 |
| C | -0.57682 | 0.4921 | -0.10428 |
| C | 0.03616 | -1.93725 | -0.04388 |
| C | -1.92761 | 0.09863 | -0.11927 |
| C | -0.17197 | 1.96816 | -0.05983 |
| C | 1.79081 | -0.07164 | -0.12016 |
| C | -1.31913 | -2.25896 | -0.03318 |
| C | 1.00027 | -2.9541 | 0.02429 |
| C | -2.28477 | -1.22209 | -0.04594 |
| H | -2.68866 | 0.84929 | -0.17681 |
| C | 1.19488 | 2.28258 | -0.04082 |
| C | -1.13714 | 2.99151 | -0.00983 |
| C | 2.16613 | 1.2443 | -0.05267 |
| H | 2.53832 | -0.8274 | -0.17359 |
| C | -1.69966 | -3.61681 | 0.02178 |
| C | 0.60758 | -4.24475 | 0.18623 |
| H | 2.04158 | -2.71487 | -0.04076 |
| C | -3.63532 | -1.55937 | 0.06456 |
| C | 1.57353 | 3.64082 | 0.01753 |
| C | -0.73959 | 4.28276 | 0.13483 |
| H | -2.18106 | 2.75701 | -0.07548 |
| C | 3.52818 | 1.58274 | 0.04466 |
| C | -3.1714 | -4.04008 | -0.15666 |
| C | -0.75818 | -4.57456 | 0.21065 |
| H | 1.34202 | -5.01614 | 0.29323 |
| C | -4.09522 | -2.83495 | 0.02629 |
| S | -4.95762 | -0.41191 | 0.31271 |
| C | 3.04932 | 4.05806 | -0.10974 |
| C | 0.62892 | 4.60478 | 0.17064 |
| H | -1.47106 | 5.06375 | 0.221 |
| C | 3.95688 | 2.86848 | 0.14633 |
| S | 4.88501 | 0.457 | 0.11355 |
| C | -3.35022 | -4.56961 | -1.5885 |
| C | -3.5374 | -5.14434 | 0.85476 |
| H | -1.05765 | -5.58944 | 0.36382 |
| C | -5.50344 | -2.94815 | 0.12736 |
| C | -6.14432 | -1.70137 | 0.12438 |
| C | 3.3051 | 4.55489 | -1.54037 |
| C | 3.38761 | 5.17734 | 0.87978 |
| H | 0.92909 | 5.62422 | 0.30718 |
| C | 5.31092 | 3.01219 | 0.48465 |
| C | 5.96051 | 1.82832 | 0.40114 |
| H | -4.36945 | -4.86619 | -1.73051 |
| H | -3.10039 | -3.80331 | -2.28923 |
| H | -2.70953 | -5.41385 | -1.73746 |
| H | -2.89803 | -5.989 | 0.71101 |
| H | -3.41128 | -4.76761 | 1.85088 |
| H | -4.55545 | -5.43792 | 0.71234 |
| H | -6.02235 | -3.88192 | 0.17324 |
| C | -7.51968 | -1.50414 | -0.03293 |
| H | 4.3356 | 4.82446 | -1.63069 |
| H | 3.0746 | 3.78138 | -2.24258 |
| H | 2.69155 | 5.40893 | -1.74044 |
| H | 2.77503 | 6.03229 | 0.68674 |
| H | 3.21511 | 4.83344 | 1.87766 |
| H | 4.41728 | 5.44058 | 0.7629 |
| H | 5.77926 | 3.9545 | 0.74618 |
| C | 7.45717 | 1.72572 | 0.48771 |
| C | -8.05183 | -0.29514 | 0.24245 |
| H | -8.14636 | -2.30408 | -0.37125 |
| C | 8.10384 | 0.5761 | 0.23 |
| H | 8.00865 | 2.59014 | 0.74304 |
| C | -7.36977 | 0.84041 | 1.01598 |
| C | -9.45832 | 0.21269 | -0.13534 |
| C | 7.52925 | -0.82251 | -0.06778 |
| C | 9.6146 | 0.43277 | 0.16272 |
| O | -6.20458 | 0.85043 | 1.47788 |
| C | -10.4007 | -0.53429 | -0.77357 |
| C | -9.50053 | 1.67921 | 0.37852 |
| O | 6.3218 | -1.12052 | -0.12222 |
| C | 10.53435 | 1.41724 | 0.28047 |
| C | 9.88063 | -1.0475 | -0.10536 |
| C | -11.6686 | -0.03409 | -1.08604 |
| C | -10.0768 | -1.86175 | -1.10958 |
| C | 11.88906 | 1.08509 | 0.27893 |
| C | 10.14293 | 2.76097 | 0.38445 |
| N | -12.7141 | 0.33764 | -1.36077 |
| N | -9.79112 | -2.94396 | -1.37408 |
| N | 12.99922 | 0.79806 | 0.27603 |
| N | 9.82359 | 3.85721 | 0.46928 |
| C | -8.42324 | 1.94284 | 1.11805 |
| S | -8.54342 | 3.46782 | 1.98973 |
| C | -10.4335 | 2.91281 | 0.25115 |
| C | 8.76029 | -1.73887 | -0.27054 |
| S | 9.07858 | -3.43631 | -0.66865 |
| C | 10.84535 | -3.08779 | -0.72714 |
| C | -10.0164 | 3.91065 | 1.08511 |
| C | -10.6896 | 5.12174 | 1.18463 |
| C | -11.4997 | 3.07339 | -0.56852 |
| C | -11.7905 | 5.3293 | 0.3459 |
| H | -10.3747 | 5.87036 | 1.88424 |
| C | -12.1834 | 4.31157 | -0.55263 |
| H | -11.8112 | 2.2878 | -1.22496 |
| C | 11.14683 | -1.85794 | -0.25948 |
| C | 12.44437 | -1.42492 | 0.00502 |
| C | 13.48651 | -2.27282 | -0.36561 |
| H | 12.63171 | -0.47553 | 0.47259 |
| C | 11.84284 | -3.91034 | -1.18709 |
| C | 13.18153 | -3.4978 | -1.01022 |
| H | 11.59982 | -4.8289 | -1.66113 |
| Cl | -13.5261 | 4.59153 | -1.6556 |
| Cl | -12.6743 | 6.84978 | 0.41384 |
| Cl | 15.15629 | -1.81901 | -0.04387 |
| Cl | 14.48977 | -4.52028 | -1.59386 |

**Table S25:** Cartesian coordinates **CTD3**

| **Atom** | **X-axis** | **Y-axis** | **Z-axis** |
| --- | --- | --- | --- |
| C | 0.43461 | -0.45837 | -0.10126 |
| C | -0.57682 | 0.4921 | -0.10428 |
| C | 0.03616 | -1.93725 | -0.04388 |
| C | -1.92761 | 0.09863 | -0.11927 |
| C | -0.17197 | 1.96816 | -0.05983 |
| C | 1.79081 | -0.07164 | -0.12016 |
| C | -1.31913 | -2.25896 | -0.03318 |
| C | 1.00027 | -2.9541 | 0.02429 |
| C | -2.28477 | -1.22209 | -0.04594 |
| H | -2.68866 | 0.84929 | -0.17681 |
| C | 1.19488 | 2.28258 | -0.04082 |
| C | -1.13714 | 2.99151 | -0.00983 |
| C | 2.16613 | 1.2443 | -0.05267 |
| H | 2.53832 | -0.8274 | -0.17359 |
| C | -1.69966 | -3.61681 | 0.02178 |
| C | 0.60758 | -4.24475 | 0.18623 |
| H | 2.04158 | -2.71487 | -0.04076 |
| C | -3.63532 | -1.55937 | 0.06456 |
| C | 1.57353 | 3.64082 | 0.01753 |
| C | -0.73959 | 4.28276 | 0.13483 |
| H | -2.18106 | 2.75701 | -0.07548 |
| C | 3.52818 | 1.58274 | 0.04466 |
| C | -3.1714 | -4.04008 | -0.15666 |
| C | -0.75818 | -4.57456 | 0.21065 |
| H | 1.34202 | -5.01614 | 0.29323 |
| C | -4.09522 | -2.83495 | 0.02629 |
| S | -4.95762 | -0.41191 | 0.31271 |
| C | 3.04932 | 4.05806 | -0.10974 |
| C | 0.62892 | 4.60478 | 0.17064 |
| H | -1.47106 | 5.06375 | 0.221 |
| C | 3.95688 | 2.86848 | 0.14633 |
| S | 4.88501 | 0.457 | 0.11355 |
| C | -3.35022 | -4.56961 | -1.5885 |
| C | -3.5374 | -5.14434 | 0.85476 |
| H | -1.05765 | -5.58944 | 0.36382 |
| C | -5.50344 | -2.94815 | 0.12736 |
| C | -6.14432 | -1.70137 | 0.12438 |
| C | 3.3051 | 4.55489 | -1.54037 |
| C | 3.38761 | 5.17734 | 0.87978 |
| H | 0.92909 | 5.62422 | 0.30718 |
| C | 5.31092 | 3.01219 | 0.48465 |
| C | 5.96051 | 1.82832 | 0.40114 |
| H | -4.36945 | -4.86619 | -1.73051 |
| H | -3.10039 | -3.80331 | -2.28923 |
| H | -2.70953 | -5.41385 | -1.73746 |
| H | -2.89803 | -5.989 | 0.71101 |
| H | -3.41128 | -4.76761 | 1.85088 |
| H | -4.55545 | -5.43792 | 0.71234 |
| H | -6.02235 | -3.88192 | 0.17324 |
| C | -7.51968 | -1.50414 | -0.03293 |
| H | 4.3356 | 4.82446 | -1.63069 |
| H | 3.0746 | 3.78138 | -2.24258 |
| H | 2.69155 | 5.40893 | -1.74044 |
| H | 2.77503 | 6.03229 | 0.68674 |
| H | 3.21511 | 4.83344 | 1.87766 |
| H | 4.41728 | 5.44058 | 0.7629 |
| H | 5.77926 | 3.9545 | 0.74618 |
| C | 7.45717 | 1.72572 | 0.48771 |
| C | -8.05183 | -0.29514 | 0.24245 |
| H | -8.14636 | -2.30408 | -0.37125 |
| C | 8.10384 | 0.5761 | 0.23 |
| H | 8.00865 | 2.59014 | 0.74304 |
| C | -7.36977 | 0.84041 | 1.01598 |
| C | -9.45832 | 0.21269 | -0.13534 |
| C | 7.52925 | -0.82251 | -0.06778 |
| C | 9.6146 | 0.43277 | 0.16272 |
| O | -6.20458 | 0.85043 | 1.47788 |
| C | -10.4007 | -0.53429 | -0.77357 |
| C | -9.50053 | 1.67921 | 0.37852 |
| O | 6.3218 | -1.12052 | -0.12222 |
| C | 10.53435 | 1.41724 | 0.28047 |
| C | 9.88063 | -1.0475 | -0.10536 |
| C | -11.6686 | -0.03409 | -1.08604 |
| C | -10.0768 | -1.86175 | -1.10958 |
| C | 11.88906 | 1.08509 | 0.27893 |
| C | 10.14293 | 2.76097 | 0.38445 |
| N | -12.7141 | 0.33764 | -1.36077 |
| N | -9.79112 | -2.94396 | -1.37408 |
| N | 12.99922 | 0.79806 | 0.27603 |
| N | 9.82359 | 3.85721 | 0.46928 |
| C | -8.42324 | 1.94284 | 1.11805 |
| S | -8.54342 | 3.46782 | 1.98973 |
| C | -10.4335 | 2.91281 | 0.25115 |
| C | 8.76029 | -1.73887 | -0.27054 |
| S | 9.07858 | -3.43631 | -0.66865 |
| C | 10.84535 | -3.08779 | -0.72714 |
| C | -10.0164 | 3.91065 | 1.08511 |
| C | -10.6896 | 5.12174 | 1.18463 |
| C | -11.4997 | 3.07339 | -0.56852 |
| C | -11.7905 | 5.3293 | 0.3459 |
| H | -10.3747 | 5.87036 | 1.88424 |
| C | -12.1834 | 4.31157 | -0.55263 |
| H | -11.8112 | 2.2878 | -1.22496 |
| C | 11.14683 | -1.85794 | -0.25948 |
| C | 12.44437 | -1.42492 | 0.00502 |
| C | 13.48651 | -2.27282 | -0.36561 |
| H | 12.63171 | -0.47553 | 0.47259 |
| C | 11.84284 | -3.91034 | -1.18709 |
| C | 13.18153 | -3.4978 | -1.01022 |
| H | 11.59982 | -4.8289 | -1.66113 |
| C | 14.94756 | -1.87573 | -0.08409 |
| N | 16.0481 | -1.57663 | 0.12796 |
| C | 14.32624 | -4.39247 | -1.52091 |
| N | 15.18849 | -5.06638 | -1.90558 |
| C | -12.5638 | 6.65972 | 0.40534 |
| N | -13.1463 | 7.66185 | 0.45012 |
| C | -13.3583 | 4.55654 | -1.51773 |
| N | -14.2432 | 4.74106 | -2.24469 |

**Table S26:** Cartesian coordinates **CTD4**

| **Atom** | **X-axis** | **Y-axis** | **Z-axis** |
| --- | --- | --- | --- |
| C | 0.50117 | 0.46605 | 0.07965 |
| C | -0.50055 | -0.46615 | -0.07945 |
| C | 0.17666 | 1.85465 | 0.27131 |
| C | -1.86356 | -0.01421 | -0.05398 |
| C | -0.17604 | -1.8547 | -0.27104 |
| C | 1.86426 | 0.01441 | 0.05418 |
| C | -1.1714 | 2.28277 | 0.25987 |
| C | 1.17796 | 2.84916 | 0.45706 |
| C | -2.19234 | 1.3193 | 0.08589 |
| H | -2.65506 | -0.7187 | -0.14812 |
| C | 1.17219 | -2.28254 | -0.25985 |
| C | -1.17749 | -2.84922 | -0.45701 |
| C | 2.19354 | -1.31912 | -0.08555 |
| H | 2.65552 | 0.71905 | 0.14781 |
| C | -1.48183 | 3.67078 | 0.3946 |
| C | 0.85183 | 4.17752 | 0.53314 |
| H | 2.19921 | 2.56547 | 0.5374 |
| C | -3.54981 | 1.74464 | 0.02146 |
| C | 1.48249 | -3.67068 | -0.39416 |
| C | -0.85146 | -4.17756 | -0.53244 |
| H | -2.19896 | -2.5655 | -0.5371 |
| C | 3.55182 | -1.74508 | -0.01988 |
| C | -2.93907 | 4.17171 | 0.48101 |
| C | -0.48763 | 4.59024 | 0.46213 |
| H | 1.62669 | 4.91213 | 0.64696 |
| C | -3.91272 | 3.04691 | 0.10937 |
| S | -4.9933 | 0.72884 | -0.24313 |
| C | 2.93989 | -4.17191 | -0.48029 |
| C | 0.48804 | -4.59013 | -0.46207 |
| H | -1.62636 | -4.9122 | -0.64631 |
| C | 3.91408 | -3.04728 | -0.10711 |
| S | 4.99616 | -0.73031 | 0.24655 |
| C | -3.22159 | 4.60051 | 1.93109 |
| C | -3.1426 | 5.37547 | -0.47529 |
| H | -0.72677 | 5.63403 | 0.47854 |
| C | -5.28105 | 3.29122 | -0.16119 |
| C | -6.03914 | 2.16826 | -0.27532 |
| C | 3.14298 | -5.37545 | 0.47542 |
| C | 3.22208 | -4.6006 | -1.93098 |
| H | 0.72717 | -5.63398 | -0.47835 |
| C | 5.28152 | -3.29236 | 0.16599 |
| C | 6.04118 | -2.1714 | 0.28452 |
| H | -4.23164 | 4.94889 | 2.00742 |
| H | -3.07965 | 3.76827 | 2.58587 |
| H | -2.55086 | 5.39025 | 2.2051 |
| H | -2.47223 | 6.16755 | -0.2124 |
| H | -2.94252 | 5.05939 | -1.48182 |
| H | -4.1518 | 5.72588 | -0.40686 |
| H | -5.68404 | 4.27736 | -0.23826 |
| C | -7.45615 | 2.23672 | -0.3578 |
| H | 4.15208 | -5.72594 | 0.40689 |
| H | 2.94283 | -5.0592 | 1.48179 |
| H | 2.47246 | -6.16734 | 0.21239 |
| H | 2.55098 | -5.39013 | -2.20497 |
| H | 3.08002 | -3.76829 | -2.586 |
| H | 4.23191 | -4.94902 | -2.00768 |
| H | 5.68458 | -4.27957 | 0.24169 |
| C | 7.45973 | -2.24123 | 0.37558 |
| C | -8.30062 | 1.1577 | -0.40449 |
| H | -7.90138 | 3.21465 | -0.3686 |
| C | 8.3031 | -1.16078 | 0.41019 |
| H | 7.90336 | -3.21858 | 0.40307 |
| C | -7.98883 | -0.35609 | -0.41904 |
| C | -9.84304 | 1.25739 | -0.45103 |
| C | 7.98439 | 0.34955 | 0.39873 |
| C | 9.84537 | -1.25918 | 0.46166 |
| O | -6.85933 | -0.91089 | -0.41971 |
| C | -10.5366 | 2.40611 | -0.59882 |
| C | -10.3801 | -0.18289 | -0.31829 |
| O | 6.85129 | 0.89761 | 0.40091 |
| C | 10.54201 | -2.40143 | 0.62084 |
| C | 10.37728 | 0.18519 | 0.30742 |
| C | -11.9352 | 2.39566 | -0.68855 |
| C | -9.83341 | 3.62106 | -0.69083 |
| C | 11.93759 | -2.39536 | 0.71544 |
| C | 9.84432 | -3.62541 | 0.71683 |
| N | -13.0804 | 2.4023 | -0.76273 |
| N | -9.25905 | 4.61343 | -0.76599 |
| N | 13.08249 | -2.40316 | 0.79438 |
| N | 9.25633 | -4.61388 | 0.79387 |
| C | -9.36799 | -1.05258 | -0.43847 |
| S | -9.91959 | -2.72186 | -0.5855 |
| C | -11.6517 | -0.79267 | -0.07783 |
| C | 9.35889 | 1.05317 | 0.3887 |
| S | 9.89453 | 2.7336 | 0.45801 |
| C | 11.56058 | 2.20093 | 0.09527 |
| C | -11.5778 | -2.19393 | -0.17743 |
| C | -12.6528 | -2.98149 | 0.03655 |
| C | -12.8646 | -0.18593 | 0.26453 |
| C | -13.8319 | -2.40062 | 0.39655 |
| H | -12.5904 | -4.04664 | -0.07611 |
| C | -13.9272 | -0.998 | 0.53765 |
| H | -12.9621 | 0.87648 | 0.33584 |
| C | 11.65153 | 0.79686 | 0.07644 |
| C | 12.88124 | 0.18714 | -0.18828 |
| C | 13.93799 | 0.99254 | -0.49095 |
| H | 12.99707 | -0.87488 | -0.17441 |
| C | 12.62766 | 2.98827 | -0.15028 |
| C | 13.81783 | 2.40319 | -0.44703 |
| H | 12.55088 | 4.05642 | -0.11417 |
| N | 14.99178 | 3.24243 | -0.73413 |
| O | 14.90213 | 4.47685 | -0.6912 |
| O | 16.06701 | 2.70748 | -1.01782 |
| N | 15.21579 | 0.38935 | -0.87967 |
| O | 16.17793 | 1.12182 | -1.15088 |
| O | 15.32215 | -0.80035 | -0.94008 |
| N | -15.1828 | -0.41044 | 0.99975 |
| O | -15.2753 | 0.81921 | 1.12596 |
| O | -16.1433 | -1.14191 | 1.27039 |
| N | -15.0169 | -3.23714 | 0.65288 |
| O | -16.094 | -2.6979 | 0.94965 |
| O | -14.9297 | -4.47086 | 0.56874 |

**Table S27:** Cartesian coordinates **CTD5**

| **Atom** | **X-axis** | **Y-axis** | **Z-axis** |
| --- | --- | --- | --- |
| C | 0.51301 | 0.47139 | 0.07349 |
| C | -0.51234 | -0.47139 | -0.07333 |
| C | 0.17383 | 1.86235 | 0.24542 |
| C | -1.87475 | -0.0342 | -0.05085 |
| C | -0.17324 | -1.86236 | -0.24524 |
| C | 1.87549 | 0.03426 | 0.05077 |
| C | -1.17335 | 2.27216 | 0.23661 |
| C | 1.18252 | 2.82432 | 0.41233 |
| C | -2.20024 | 1.29308 | 0.07245 |
| H | -2.65984 | -0.76491 | -0.14181 |
| C | 1.17399 | -2.2722 | -0.23644 |
| C | -1.18204 | -2.82431 | -0.41198 |
| C | 2.2011 | -1.29317 | -0.07201 |
| H | 2.66047 | 0.76509 | 0.14177 |
| C | -1.47667 | 3.65156 | 0.36375 |
| C | 0.86435 | 4.13662 | 0.47539 |
| H | 2.21203 | 2.52176 | 0.49063 |
| C | -3.56143 | 1.73682 | 0.01707 |
| C | 1.47717 | -3.6516 | -0.3634 |
| C | -0.86394 | -4.13661 | -0.47503 |
| H | -2.21155 | -2.52176 | -0.49034 |
| C | 3.56243 | -1.73731 | -0.01616 |
| C | -2.92775 | 4.15796 | 0.48081 |
| C | -0.47507 | 4.54944 | 0.41102 |
| H | 1.6379 | 4.87682 | 0.57465 |
| C | -3.90884 | 3.055 | 0.08888 |
| S | -5.01438 | 0.72612 | -0.20459 |
| C | 2.92819 | -4.15814 | -0.48032 |
| C | 0.47548 | -4.54944 | -0.41068 |
| H | -1.63752 | -4.87683 | -0.57408 |
| C | 3.90936 | -3.05572 | -0.08768 |
| S | 5.01572 | -0.72756 | 0.20631 |
| C | -3.20117 | 4.54711 | 1.9444 |
| C | -3.13665 | 5.38915 | -0.4246 |
| H | -0.71371 | 5.58234 | 0.41388 |
| C | -5.25984 | 3.31912 | -0.21541 |
| C | -6.05107 | 2.16288 | -0.3063 |
| C | 3.13693 | -5.38934 | 0.42484 |
| C | 3.20157 | -4.5471 | -1.94408 |
| H | 0.7141 | -5.58232 | -0.41356 |
| C | 5.25943 | -3.32071 | 0.21897 |
| C | 6.05033 | -2.1655 | 0.31191 |
| H | -4.20981 | 4.89497 | 2.03189 |
| H | -3.05971 | 3.70054 | 2.58099 |
| H | -2.52745 | 5.32718 | 2.23382 |
| H | -2.46224 | 6.16542 | -0.13427 |
| H | -2.94998 | 5.1201 | -1.44659 |
| H | -4.14424 | 5.73312 | -0.32745 |
| H | -5.64414 | 4.31138 | -0.33071 |
| C | -7.45849 | 2.23831 | -0.41645 |
| H | 4.14447 | -5.73337 | 0.32773 |
| H | 2.95024 | -5.12032 | 1.44689 |
| H | 2.46244 | -6.16549 | 0.13448 |
| H | 2.52769 | -5.32709 | -2.23351 |
| H | 3.06012 | -3.70048 | -2.58058 |
| H | 4.21012 | -4.89509 | -2.03167 |
| H | 5.6438 | -4.31305 | 0.33326 |
| C | 7.45704 | -2.24154 | 0.42611 |
| C | -8.29439 | 1.16057 | -0.42447 |
| H | -7.90463 | 3.21138 | -0.48658 |
| C | 8.29211 | -1.16311 | 0.42815 |
| H | 7.90369 | -3.21334 | 0.50386 |
| C | -7.96416 | -0.34697 | -0.40142 |
| C | -9.83969 | 1.25103 | -0.44809 |
| C | 7.95855 | 0.34349 | 0.3901 |
| C | 9.83798 | -1.25087 | 0.45621 |
| O | -6.82495 | -0.87781 | -0.38712 |
| C | -10.5514 | 2.39533 | -0.57234 |
| C | -10.3579 | -0.19688 | -0.31269 |
| O | 6.81563 | 0.87205 | 0.37331 |
| C | 10.5526 | -2.3941 | 0.58711 |
| C | 10.35109 | 0.19552 | 0.3125 |
| C | -11.9507 | 2.37324 | -0.63579 |
| C | -9.85697 | 3.61185 | -0.65258 |
| C | 11.95022 | -2.37186 | 0.6531 |
| C | 9.85829 | -3.61148 | 0.66871 |
| N | -13.094 | 2.3648 | -0.6883 |
| N | -9.277 | 4.6006 | -0.71704 |
| N | 13.09227 | -2.36182 | 0.70788 |
| N | 9.27766 | -4.59946 | 0.73387 |
| C | -9.3366 | -1.06164 | -0.41216 |
| S | -9.87729 | -2.73834 | -0.5182 |
| C | -11.6268 | -0.80734 | -0.08642 |
| C | 9.33001 | 1.06047 | 0.39067 |
| S | 9.86455 | 2.74172 | 0.46239 |
| C | 11.52409 | 2.20001 | 0.10145 |
| C | -11.5364 | -2.20385 | -0.13634 |
| C | -12.6474 | -3.01036 | 0.10562 |
| C | -12.8536 | -0.1923 | 0.20359 |
| C | -13.8311 | -2.41154 | 0.42306 |
| H | -12.575 | -4.07586 | 0.03978 |
| C | -13.9323 | -0.99563 | 0.49917 |
| H | -12.9475 | 0.87361 | 0.20875 |
| C | 11.61947 | 0.80424 | 0.09139 |
| C | 12.84889 | 0.18265 | -0.15986 |
| C | 13.9492 | 0.99941 | -0.48314 |
| H | 12.94501 | -0.88369 | -0.12028 |
| C | 12.63704 | 3.01011 | -0.16194 |
| C | 13.83306 | 2.41606 | -0.45027 |
| H | 12.55174 | 4.07493 | -0.13531 |
| C | 15.2968 | 0.36693 | -0.89186 |
| C | 15.06829 | 3.29389 | -0.74996 |
| C | -15.0726 | -3.28522 | 0.70868 |
| C | -15.2747 | -0.36754 | 0.93632 |
| F | -15.2863 | -0.22937 | 2.28064 |
| F | -15.4204 | 0.84291 | 0.36034 |
| F | -14.9893 | -4.43142 | 0.00005 |
| F | -15.1278 | -3.57467 | 2.02525 |
| F | -16.1891 | -2.61634 | 0.34919 |
| F | -16.2934 | -1.17044 | 0.56133 |
| F | 15.32832 | 0.2129 | -2.23498 |
| F | 16.30899 | 1.17967 | -0.51675 |
| F | 15.44201 | -0.83498 | -0.29656 |
| F | 15.87758 | 2.65372 | -1.6198 |
| F | 15.73999 | 3.5319 | 0.39752 |
| F | 14.66829 | 4.46546 | -1.28657 |

**Table S28:** Cartesian coordinates **CTD6**

| **Atom** | **X-axis** | **Y-axis** | **Z-axis** |
| --- | --- | --- | --- |
| C | 0.51464 | 0.47903 | 0.09216 |
| C | -0.51473 | -0.47916 | -0.09195 |
| C | 0.18018 | 1.85255 | 0.29274 |
| C | -1.87133 | -0.0265 | -0.08208 |
| C | -0.18029 | -1.85271 | -0.29245 |
| C | 1.87124 | 0.02633 | 0.08222 |
| C | -1.17349 | 2.26973 | 0.27207 |
| C | 1.17749 | 2.84174 | 0.49176 |
| C | -2.19609 | 1.30342 | 0.08898 |
| H | -2.65725 | -0.73595 | -0.20867 |
| C | 1.17332 | -2.2699 | -0.27185 |
| C | -1.17758 | -2.84191 | -0.49134 |
| C | 2.19591 | -1.3036 | -0.08878 |
| H | 2.65721 | 0.73576 | 0.20874 |
| C | -1.48576 | 3.65465 | 0.41668 |
| C | 0.84748 | 4.16298 | 0.59677 |
| H | 2.20183 | 2.55228 | 0.56492 |
| C | -3.55284 | 1.72643 | 0.03629 |
| C | 1.48557 | -3.65487 | -0.41636 |
| C | -0.84758 | -4.16319 | -0.59621 |
| H | -2.20191 | -2.55246 | -0.56445 |
| C | 3.55259 | -1.7267 | -0.03598 |
| C | -2.9441 | 4.1618 | 0.47364 |
| C | -0.49046 | 4.57316 | 0.51668 |
| H | 1.61776 | 4.89703 | 0.73804 |
| C | -3.91714 | 3.03303 | 0.13543 |
| S | -4.99244 | 0.70532 | -0.25034 |
| C | 2.94383 | -4.16201 | -0.4734 |
| C | 0.49034 | -4.57336 | -0.51623 |
| H | -1.61784 | -4.89724 | -0.73735 |
| C | 3.91668 | -3.03332 | -0.13516 |
| S | 4.99242 | -0.70589 | 0.25096 |
| C | -3.2427 | 4.6597 | 1.90118 |
| C | -3.14066 | 5.31677 | -0.52351 |
| H | -0.73043 | 5.61938 | 0.55939 |
| C | -5.29144 | 3.28271 | -0.08306 |
| C | -6.06582 | 2.12231 | -0.22238 |
| C | 3.14062 | -5.31688 | 0.52381 |
| C | 3.24246 | -4.65998 | -1.90092 |
| H | 0.73035 | -5.61958 | -0.55884 |
| C | 5.29062 | -3.2833 | 0.08357 |
| C | 6.06525 | -2.12346 | 0.22362 |
| H | -4.25461 | 5.01178 | 1.94767 |
| H | -3.10965 | 3.86522 | 2.59969 |
| H | -2.57633 | 5.46392 | 2.14175 |
| H | -2.47491 | 6.11689 | -0.28748 |
| H | -2.93314 | 4.95986 | -1.51455 |
| H | -4.15045 | 5.66473 | -0.47633 |
| H | -5.70689 | 4.26739 | -0.11144 |
| C | -7.48164 | 2.19244 | -0.29673 |
| H | 4.15045 | -5.66474 | 0.47655 |
| H | 2.9332 | -4.95988 | 1.51486 |
| H | 2.47493 | -6.11709 | 0.28797 |
| H | 2.57615 | -5.46428 | -2.14144 |
| H | 3.10936 | -3.86557 | -2.59948 |
| H | 4.2544 | -5.01202 | -1.94739 |
| H | 5.70591 | -4.26804 | 0.1115 |
| C | 7.48126 | -2.19571 | 0.2991 |
| C | -8.33283 | 1.1282 | -0.38257 |
| H | -7.92192 | 3.16755 | -0.27436 |
| C | 8.33448 | -1.13358 | 0.38447 |
| H | 7.9196 | -3.1711 | 0.27711 |
| C | -8.02281 | -0.38402 | -0.40373 |
| C | -9.87859 | 1.24789 | -0.47435 |
| C | 8.02613 | 0.37662 | 0.40326 |
| C | 9.88191 | -1.25503 | 0.47706 |
| O | -6.89076 | -0.93091 | -0.40858 |
| C | -10.5643 | 2.41512 | -0.66852 |
| C | -10.4134 | -0.20266 | -0.31478 |
| O | 6.89428 | 0.926 | 0.40981 |
| C | 10.56486 | -2.42125 | 0.67154 |
| C | 10.4222 | 0.193 | 0.31475 |
| C | -11.9623 | 2.44561 | -0.79917 |
| C | -9.84795 | 3.62276 | -0.76982 |
| C | 11.96162 | -2.44764 | 0.80106 |
| C | 9.84721 | -3.62717 | 0.77215 |
| N | -13.1043 | 2.48965 | -0.90825 |
| N | -9.26339 | 4.60825 | -0.85249 |
| N | 13.10338 | -2.48652 | 0.90916 |
| N | 9.2599 | -4.61116 | 0.85411 |
| C | -9.40029 | -1.07952 | -0.42053 |
| S | -9.95182 | -2.75357 | -0.53727 |
| C | -11.6794 | -0.81404 | -0.0662 |
| C | 9.40449 | 1.06975 | 0.41383 |
| S | 9.9457 | 2.74417 | 0.51534 |
| C | 11.59216 | 2.20915 | 0.09963 |
| C | -11.5978 | -2.21169 | -0.12313 |
| C | -12.7066 | -3.01467 | 0.13103 |
| C | -12.8925 | -0.20116 | 0.25908 |
| C | -13.8786 | -2.4251 | 0.46233 |
| H | -12.6431 | -4.08077 | 0.07445 |
| C | -13.9756 | -1.00879 | 0.54945 |
| H | -12.9757 | 0.86468 | 0.29088 |
| C | 11.69434 | 0.8076 | 0.06809 |
| C | 12.93157 | 0.2073 | -0.23651 |
| C | 13.99359 | 1.0443 | -0.55095 |
| H | 13.04399 | -0.85732 | -0.23763 |
| C | 12.68283 | 3.02606 | -0.17452 |
| C | 13.86342 | 2.44696 | -0.48474 |
| H | 12.5986 | 4.09113 | -0.14463 |
| S | -15.5188 | -0.28813 | 1.05072 |
| S | -15.2804 | -3.49082 | 0.77077 |
| S | 15.58245 | 0.43427 | -1.06893 |
| S | 15.28236 | 3.48497 | -0.78591 |
| O | -16.4096 | -3.07818 | -0.07522 |
| O | -15.7431 | -3.41234 | 2.37867 |
| O | -14.8972 | -4.87041 | 0.44358 |
| O | -16.494 | -1.35861 | 1.30233 |
| O | -15.3143 | 0.48865 | 2.28004 |
| O | -16.0914 | 0.71269 | -0.15555 |
| O | 14.90128 | 4.8866 | -0.57534 |
| O | 15.83983 | 3.29465 | -2.34785 |
| O | 16.35436 | 3.11438 | 0.14802 |
| O | 15.41928 | -0.4956 | -2.1919 |
| O | 16.40452 | 1.57992 | -1.49584 |
| O | 16.33415 | -0.35545 | 0.19189 |
| H | -16.2257 | 0.20194 | -0.95752 |
| H | -15.0215 | -3.71176 | 2.94092 |
| H | 16.61715 | 3.85084 | -2.47167 |
| H | 17.18071 | -0.6953 | -0.11037 |

**Table S29:** Cartesian coordinates **CTD7**

| **Atom** | **X-axis** | **Y-axis** | **Z-axis** |
| --- | --- | --- | --- |
| C | 0.51101 | 0.46812 | 0.07427 |
| C | -0.50959 | -0.46829 | -0.07392 |
| C | 0.16843 | 1.81232 | 0.22687 |
| C | -1.87406 | -0.06711 | -0.0631 |
| C | -0.16669 | -1.81194 | -0.2287 |
| C | 1.87593 | 0.06709 | 0.0642 |
| C | -1.18279 | 2.21622 | 0.22626 |
| C | 1.18096 | 2.75799 | 0.37925 |
| C | -2.21431 | 1.25289 | 0.07279 |
| H | -2.64241 | -0.80419 | -0.17237 |
| C | 1.18574 | -2.21596 | -0.22686 |
| C | -1.1802 | -2.7577 | -0.37992 |
| C | 2.21877 | -1.25282 | -0.0707 |
| H | 2.64306 | 0.80597 | 0.17161 |
| C | -1.47651 | 3.59324 | 0.35937 |
| C | 0.87337 | 4.07191 | 0.44602 |
| H | 2.20163 | 2.44118 | 0.43748 |
| C | -3.57561 | 1.68607 | 0.03507 |
| C | 1.47923 | -3.59247 | -0.35716 |
| C | -0.87287 | -4.07172 | -0.44659 |
| H | -2.2014 | -2.44141 | -0.43592 |
| C | 3.58253 | -1.6837 | -0.02887 |
| C | -2.927 | 4.11302 | 0.48036 |
| C | -0.46462 | 4.49448 | 0.40216 |
| H | 1.65581 | 4.79403 | 0.53898 |
| C | -3.91803 | 3.00587 | 0.09495 |
| S | -5.03869 | 0.67823 | -0.15816 |
| C | 2.93089 | -4.11239 | -0.47816 |
| C | 0.46524 | -4.49401 | -0.40141 |
| H | -1.65582 | -4.79407 | -0.53783 |
| C | 3.92631 | -3.00311 | -0.0953 |
| S | 5.04573 | -0.67274 | 0.16484 |
| C | -3.19013 | 4.51469 | 1.94386 |
| C | -3.12791 | 5.35324 | -0.42409 |
| H | -0.68924 | 5.53855 | 0.41814 |
| C | -5.27357 | 3.27318 | -0.20581 |
| C | -6.07286 | 2.12052 | -0.29038 |
| C | 3.12903 | -5.35333 | 0.42406 |
| C | 3.19169 | -4.51404 | -1.94369 |
| H | 0.68964 | -5.53833 | -0.41786 |
| C | 5.28732 | -3.26864 | 0.20418 |
| C | 6.0902 | -2.1128 | 0.29051 |
| H | -4.19469 | 4.87559 | 2.03678 |
| H | -3.05484 | 3.66882 | 2.58134 |
| H | -2.50672 | 5.28937 | 2.22851 |
| H | -2.44365 | 6.12385 | -0.13332 |
| H | -2.94759 | 5.08392 | -1.44471 |
| H | -4.13132 | 5.71217 | -0.32241 |
| H | -5.6686 | 4.2584 | -0.32567 |
| C | -7.49462 | 2.20876 | -0.42 |
| H | 4.13157 | -5.71347 | 0.32382 |
| H | 2.94793 | -5.08406 | 1.44545 |
| H | 2.44332 | -6.12237 | 0.1331 |
| H | 2.5064 | -5.28851 | -2.22655 |
| H | 3.05552 | -3.6684 | -2.58056 |
| H | 4.19507 | -4.87615 | -2.03756 |
| H | 5.68085 | -4.25413 | 0.33374 |
| C | 7.51825 | -2.19802 | 0.42274 |
| C | -8.35977 | 1.14991 | -0.4349 |
| H | -7.91722 | 3.18381 | -0.49893 |
| C | 8.38912 | -1.13765 | 0.43506 |
| H | 7.93802 | -3.17011 | 0.51468 |
| C | -8.03276 | -0.35365 | -0.41328 |
| C | -9.91267 | 1.25278 | -0.46798 |
| C | 8.08952 | 0.36972 | 0.44711 |
| C | 9.93198 | -1.24603 | 0.44714 |
| O | -6.90409 | -0.89883 | -0.39472 |
| C | -10.5999 | 2.40183 | -0.57656 |
| C | -10.4315 | -0.20485 | -0.36407 |
| O | 6.97062 | 0.9398 | 0.43462 |
| C | 10.60882 | -2.41477 | 0.58337 |
| C | 10.468 | 0.21222 | 0.2929 |
| C | -11.9962 | 2.37942 | -0.64253 |
| C | -9.88533 | 3.61728 | -0.64864 |
| C | 11.99309 | -2.40712 | 0.64828 |
| C | 9.89557 | -3.62955 | 0.66402 |
| N | -13.1512 | 2.38381 | -0.69969 |
| N | -9.30837 | 4.5797 | -0.70559 |
| N | 13.15349 | -2.39926 | 0.70255 |
| N | 9.29928 | -4.60804 | 0.72741 |
| C | -9.38921 | -1.05888 | -0.43475 |
| S | -9.87716 | -2.73042 | -0.54193 |
| C | -11.7003 | -0.84514 | -0.16906 |
| C | 9.45098 | 1.0646 | 0.4738 |
| S | 9.95344 | 2.7354 | 0.67744 |
| C | 11.58249 | 2.26453 | 0.15526 |
| C | -11.5301 | -2.22794 | -0.13789 |
| C | -12.5476 | -3.08012 | 0.19269 |
| C | -12.9917 | -0.30961 | 0.01269 |
| C | -13.7448 | -2.57337 | 0.53732 |
| H | -12.388 | -4.13932 | 0.18533 |
| C | -13.997 | -1.17803 | 0.38693 |
| H | -13.2007 | 0.74093 | -0.11311 |
| C | 11.70751 | 0.87314 | -0.00137 |
| C | 12.93025 | 0.33379 | -0.42883 |
| C | 13.92536 | 1.17033 | -0.78779 |
| H | 13.10283 | -0.70871 | -0.49684 |
| C | 12.63635 | 3.12281 | -0.10211 |
| C | 13.77649 | 2.585 | -0.6517 |
| H | 12.54216 | 4.17214 | 0.11884 |
| O | -15.8347 | -3.34941 | 1.95139 |
| O | -14.5092 | -4.81679 | 0.7571 |
| O | -16.2767 | -1.37308 | 1.17564 |
| O | -15.7649 | 0.65224 | 0.15732 |
| O | 14.68112 | 4.76682 | -1.12003 |
| O | 16.07887 | 2.98071 | -1.75894 |
| O | 15.30255 | -0.60816 | -1.93126 |
| O | 16.27714 | 1.24978 | -1.35803 |
| C | -15.4174 | -0.64789 | 0.61256 |
| C | -14.7339 | -3.62853 | 1.08291 |
| C | 14.87548 | 3.52457 | -1.17485 |
| C | 15.23249 | 0.55944 | -1.38617 |
| C | 16.98099 | 4.04828 | -2.04058 |
| H | 17.71826 | 3.7377 | -2.76413 |
| H | 16.40168 | 4.84351 | -2.43758 |
| H | 17.48639 | 4.36727 | -1.15315 |
| C | -16.3405 | 0.41958 | -1.13256 |
| H | -17.1939 | -0.21322 | -1.01471 |
| H | -16.6395 | 1.34446 | -1.57994 |
| H | -15.62 | -0.06168 | -1.76288 |
| C | -15.2367 | -3.68774 | 3.21697 |
| H | -14.3896 | -3.05362 | 3.35713 |
| H | -15.923 | -3.55596 | 4.03753 |
| H | -14.9269 | -4.71698 | 3.1851 |
| C | 16.59257 | -0.98419 | -2.48418 |
| H | 17.15029 | -1.54805 | -1.75188 |
| H | 16.47148 | -1.59015 | -3.36722 |
| H | 17.12241 | -0.08855 | -2.73322 |

**References**

1. Doust Mohammadi, M. *et al.* Increasing the Photovoltaic Power of the Organic Solar Cells by Structural Modification of the R-P2F-Based Materials. *J Mol Model* **29**, 1–18 (2023).

2. Khalid, M. *et al.* Effect of different end-capped donor moieties on non-fullerenes based non-covalently fused-ring derivatives for achieving high-performance NLO properties. *Sci Rep* **13**, 1395 (2023).

3. Zhan, C.-G., Nichols, J. A. & Dixon, D. A. Ionization Potential, Electron Affinity, Electronegativity, Hardness, and Electron Excitation Energy: Molecular Properties from Density Functional Theory Orbital Energies. *J. Phys. Chem. A* **107**, 4184–4195 (2003).

4. Koopmans, T. Ordering of wave functions and eigenenergies to the individual electrons of an atom. *Physica* **1**, 104–113 (1933).

5. Serdaroğlu, G. & Mustafa, E. A Computational study predicting the chemical reactivity behavior of 1-substituted 9-ethyl-βCCM derivatives: DFT-Based Quantum Chemical Descriptors. *Turkish Computational and Theoretical Chemistry* **2**, 1–11 (2018).
